# Artesunate Inhibits Growth of Sunitinib-Resistant Renal Cell Carcinoma Cells through Cell Cycle Arrest and Induction of Ferroptosis

**DOI:** 10.3390/cancers12113150

**Published:** 2020-10-27

**Authors:** Sascha D. Markowitsch, Patricia Schupp, Julia Lauckner, Olesya Vakhrusheva, Kimberly S. Slade, René Mager, Thomas Efferth, Axel Haferkamp, Eva Juengel

**Affiliations:** 1 Department of Urology and Pediatric Urology, University Medical Center Mainz, Langenbeckstraße 1, 55131 Mainz, Germany; sascha.markowitsch@unimedizin-mainz.de (S.D.M.); pschupp@students.uni-mainz.de (P.S.); jlauckne@students.uni-mainz.de (J.L.); olesya.vakhrusheva@unimedizin-mainz.de (O.V.); kimberlysue.slade@unimedizin-mainz.de (K.S.S.); rene.mager@unimedizin-mainz.de (R.M.); axel.haferkamp@unimedizin-mainz.de (A.H.); 2 Institute of Pharmaceutical and Biomedical Sciences, Johannes Gutenberg University Mainz, Staudingerweg 5, 55128 Mainz, Germany; efferth@uni-mainz.de

**Keywords:** renal cell carcinoma (RCC), sunitib resistance, artesunate (ART), Traditional Chinese Medicine (TCM), growth inhibition, ferroptosis, reactive oxygen species (ROS)

## Abstract

**Simple Summary:**

Renal cell carcinoma (RCC) is the most common kidney malignancy. Due to development of therapy resistance, efficacy of conventional drugs such as sunitinib is limited. Artesunate (ART), a drug originating from Traditional Chinese Medicine, has exhibited anti-tumor effects in several non-urologic tumors. ART inhibited growth, reduced metastatic properties, and curtailed metabolism in sunitinib-sensitive and sunitinib–resistant RCC cells. In three of four tested cell lines, ART’s growth inhibitory effects were accompanied by cell cycle arrest and modulation of cell cycle regulating proteins. In a fourth cell line, KTCTL-26, ART evoked ferroptosis, an iron-dependent cell death, and exhibited stronger anti-tumor effects than in the other cell lines. The regulatory protein, p53, was only detectable in the KTCTL-26 cells, possibly making p53 a predictive marker of cancer that may respond better to ART. ART, therefore, may hold promise as an additive therapy option for selected patients with advanced or therapy-resistant RCC.

**Abstract:**

Although innovative therapeutic concepts have led to better treatment of advanced renal cell carcinoma (RCC), efficacy is still limited due to the tumor developing resistance to applied drugs. Artesunate (ART) has demonstrated anti-tumor effects in different tumor entities. This study was designed to investigate the impact of ART (1–100 µM) on the sunitinib-resistant RCC cell lines, Caki-1, 786-O, KTCTL26, and A-498. Therapy-sensitive (parental) and untreated cells served as controls. ART’s impact on tumor cell growth, proliferation, clonogenic growth, apoptosis, necrosis, ferroptosis, and metabolic activity was evaluated. Cell cycle distribution, the expression of cell cycle regulating proteins, p53, and the occurrence of reactive oxygen species (ROS) were investigated. ART significantly increased cytotoxicity and inhibited proliferation and clonogenic growth in both parental and sunitinib-resistant RCC cells. In Caki-1, 786-O, and A-498 cell lines growth inhibition was associated with G0/G1 phase arrest and distinct modulation of cell cycle regulating proteins. KTCTL-26 cells were mainly affected by ART through ROS generation, ferroptosis, and decreased metabolism. p53 exclusively appeared in the KTCTL-26 cells, indicating that p53 might be predictive for ART-dependent ferroptosis. Thus, ART may hold promise for treating selected patients with advanced and even therapy-resistant RCC.

## 1. Introduction

Accounting for ~85% of cases, renal cell carcinoma (RCC) is the most common kidney cancer and one of the most aggressive urologic cancers [1]. At initial diagnosis, RCC patients often present at an advanced stage with an accordingly poor prognosis [2]. Better understanding of the molecular modes of action underlying RCC led to the development of targeted therapies affecting angiogenic activity and immune checkpoint inhibitors. However, due to the development of resistance, the efficacy of even these targeted treatments is limited. Since RCC is an angiogenic disease, a promising avenue of treatment is to block angiogenesis, thereby suppressing the supply of oxygen and nutrients to the tumor. Initially, the anti-angiogenic activity of the tyrosine kinase inhibitor (TKI) sunitinib extends the progression-free survival of patients [3], but resistance occurs during treatment [4]. Thus, therapy resistance is one, if not the main, problem, in treating advanced RCC. Novel treatment strategies combining targeted therapy and immunotherapy have been introduced [5,6,7]. However, even with combined drug application resistance occurs and adverse side effects are common [5,8,9]. 

Certainly, in part due to the long-term curative failure of conventional therapy, the demand for traditional and alternative medicine is growing worldwide [10,11]. Patients hope that complementary therapeutic approaches would increase effectiveness and/or reduce side effects [12,13], and 40–50% of European cancer patients indeed use complementary and alternative therapies [14,15,16]. However, solid and reliable studies with regard to natural substances and their derivatives are sparse and the lack of proven efficacy coupled with uncoordinated self-treatment is perilous. Contraindications as well as adverse side effects of herbal compounds combined with conventional therapy also cannot be ignored [17].

Some studies have been carried out indicating anti-tumor effects of natural compounds and their derivatives, especially if applied together with established therapies or by counteracting therapy resistance [18,19,20,21]. Artemisinin from the annual mugwort (*Artemisia annua*) has been used in Traditional Chinese Medicine for over 2000 years, particularly in treating malaria [22] and is still in use. An anti-tumor effect of the artemisinin derivative, artesunate (ART), was reported in 2001 [23]. Subsequently, anti-tumor effects of ART in vitro and in vivo were reported in different tumor entities, including therapy-resistant tumors, with fewer side effects in combination with conventional therapy [23,24,25,26,27]. ART, a semi-synthetic water-soluble derivative of artemisinin, exhibits hydrophilic properties in contrast to the natural substance, and has better bioavailability and anti-tumor activity than artemisinin [28,29]. In therapy-sensitive and doxorubicin-resistant T-leukemia cells, ART induced apoptosis and displayed a synergistic effect in combination with doxorubicin [30]. ART’s anti-tumor effect had also been demonstrated in gastrointestinal [31] and breast cancer [32]. Here, ART specifically affected neoplastic tissue and spares healthy tissue [33]. 

The mode of action of ART is not yet fully understood. Both in the malaria pathogen and in tumor cells, high cellular iron content seems to play a role in the response to ART (and artemisinin) [34]. A high iron content facilitates ferroptosis, an iron-dependent cell death caused by reactive oxygen species (ROS) formation [34,35,36,37,38]. Iron has been identified as central player in cancer progression [39]. This could explain why artemisinin and ART specifically affect tumor cells but not normal cells with lower iron content [33]. Correspondingly, RCC cells express significantly more iron-regulated genes [40]. Furthermore, RCC tissue, particularly clear cell RCC tissue, compared to healthy tissue, exhibits significant higher transferrin receptor 1 (TfR1) expression, which is responsible for iron uptake and associated with worse survival outcomes [41]. Moreover, artemisinin and its derivatives inhibit angiogenesis [42,43,44,45]. ART reduced the expression of angiogenic proteins in hemangioendothelioma cells, and thus has been postulated to be a therapeutic option for angiogenic cancers [46], including RCC. Indeed, ART exerted potent, selective cytotoxicity in therapy-sensitive RCC [47] and inhibited invasiveness in vitro and in vivo. It induced ferroptosis in therapy-sensitive RCC cells [48] and enhanced the anti-tumor effect of the TKI sorafenib [47].

Investigations exploring the effect of ART on therapy-sensitive RCC are scant and unavailable in regard to therapy-resistant RCC. Thus, the present study was designed to evaluate ART’s impact on sensitive and more importantly sunitinib-resistant RCC cells, by evaluating the effect of this drug on tumor growth and underlying molecular mechanisms. The intent of this study was to implement rationale for a founded treatment option with a compound of natural origin for patients with advanced and/or therapy-resistant RCC.

## 2. Results

### 2.1. Confirmation of Sunitinib Resistance in RCC Cells

Sunitinib-sensitive and sunitinib-resistant RCC cell lines, Caki-1, 786-O, KTCTL-26, and A-498, were employed with the sunitinib-sensitive (parental) RCC sub-lines serving as controls. Cells were designated sunitinib-resistant if the IC50 under escalating sunitinib dosage (0.1–100 µM) was approximately twice as high as the IC50 of the sunitinib-sensitive counterpart. Even though the A-498 cells barely reached the designated IC50 (IC50 of 19.30 µM in the resistant cells compared to 10.43 µM in their parental counterparts), all four cell lines fulfilled this specification (Table 1). Here, with an IC50 of 10.43 µM, parental A-498 revealed the weakest sunitinib response, compared to the other three RCC cell lines. The most prominent difference in IC50 was found in Caki-1 cells with an IC50 of 2.58 µM in parental and 19.13 µM in resistant cells, indicating that initial high sensitivity may lead to stronger resistance development in RCC cells. The differences in IC50 for the parental and resistant 786-O and KTCTL-26 cell lines lay between those of the A-498 and Caki-1cells. The IC50 of parental 786-O of 3.97 µM elevated in the resistant 786-O to an IC50 of 11.16 µM. In KTCTL-26 cells, IC50 of 6.37 µM in the parental cells increased to an IC50 of 13.31 µM in the resistant counterparts.

### 2.2. Artesunate Inhibits Cell Growth of Parental and Sunitinib-Resistant RCC Cells

**Figure 1:**
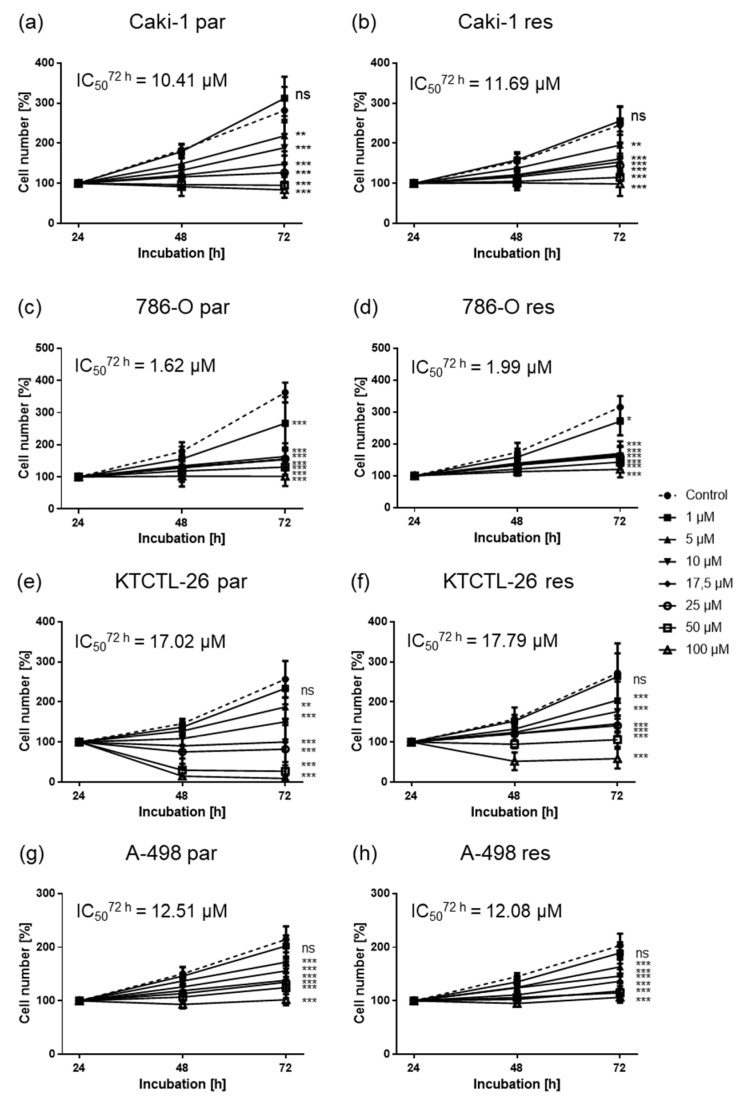
Tumor cell growth after exposure to artesunate (ART): Tumor cell growth of parental (par) and sunitinib-resistant (res) Caki-1 (**a**,**b**), 786-O (**c**,**d**), KTCTL-26 (**e**,**f**), A-498 (**g**,**h**) cells after 24, 48, and 72 h treatment with ascending ART concentrations (1–100 µM). Cell number set to 100% after 24 h incubation. The IC50 of ART after 72 h treatment is specified. Error bars indicate standard deviation (*SD*). Significant difference to untreated control: * *p* ≤ 0.05, ** *p* ≤ 0.01, *** *p* ≤ 0.001, ns = not significant. *n* = 5.

ART induced a dose- and time-dependent growth inhibition in all parental and resistant RCC cell lines, compared to the untreated controls (Figure 1), with comparable IC50 values for corresponding parental and resistant sub-lines. A significant growth reduction of parental Caki-1 cells with an IC50 of 10.41 µM ART after 72 h was apparent (Figure 1a). The sunitinib-resistant Caki-1 cells were similarly inhibited with an IC50 of 11.69 µM ART after 72 h treatment (Figure 1b). In both parental and sunitinib-resistant Caki-1 cells, significant growth inhibition was first reached with 5 µM ART (Figure 1a,b). Parental and sunitinib-resistant A-498 cells also first showed significant growth inhibition at a concentration of 5 µM ART, with an IC50 of 12.51 µM ART for the parental and 12.08 µM ART for the sunitinib-resistant cells (Figure 1g,h). The most prominent growth inhibition was found in the 786-O cell lines, with an IC50 of 1.62 µM ART in the parental and an IC50 of 1.99 µM ART in the resistant 786-O cells (Figure 1c,d). In 786-O cells, exposure to 1 µM ART already resulted in significant growth inhibition that further increased with ascending concentration (Figure 1c,d). In all three of the abovementioned RCC cell lines, tumor cell growth was arrested but not reduced below the initially seeded basal cell count, even at the highest concentration of 100 µM ART (Figure 1a–d,g,h). In KTCTL-26 a significant decrease in parental and sunitinib-resistant cells, below the initial seeding cell count (Figure 1e,f), did take place. ART’s IC50 values for the KTCTL-26 cell lines were higher (parental: IC50 = 17.02 µM, sunitinib-resistant: IC50 = 17.79 µM) than those of the other cell lines. Comparable to Caki-1 and A-498 cells, the first significant inhibitory response in regard to KTCTL-26 growth was detected after exposure to 5 µM ART (Figure 1e,f). In summary, ART treatment significantly suppressed tumor cell growth in all four RCC cell lines, but only in the KTCTL-26 cell line did growth reduction result in a decrease of cell counts below the number of initially seeded cells.

### 2.3. Artesunate Impairs RCC Cell Proliferation

**Figure 2:**
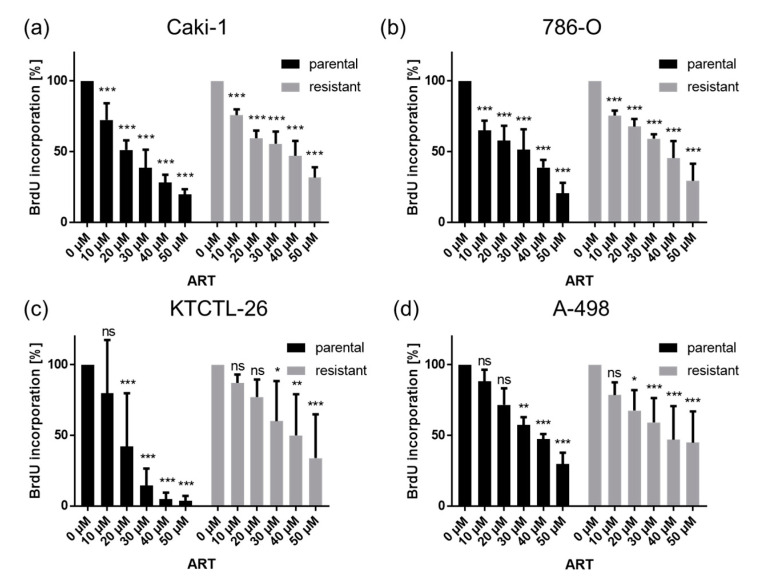
Cell proliferation: Tumor cell proliferation of parental (par) and sunitinib-resistant Caki-1 (**a**), 786-O (**b**), KTCTL-26 (**c**), and A-498 (**d**) RCC cells incubated for 72 h with ART (10–50 µM). Untreated controls were set to 100%. Error bars indicate standard deviation (*SD*). Significant difference to untreated control: * *p* ≤ 0.05, ** *p* ≤ 0.01, *** *p* ≤ 0.001, ns = not significant. *n* = 5.

Exposure to ART for 72 h contributed to significant dose-dependent inhibition of RCC cell proliferation (Figure 2). The proliferation of parental and sunitinib-resistant Caki-1 and 786-O cells was already significantly reduced after treatment with 10 µM ART, compared to the untreated controls (Figure 2a,b). Parental KTCTL-26 cells revealed a significant proliferation inhibition after exposure to 20 µM ART, while resistant KTCTL-26 cells were significantly inhibited at 30 µM ART (Figure 2c). A-498 cells behaved differently in respect to the inhibiting concentration of ART. Proliferation of the resistant A-498 cells was already significantly reduced after treatment with 20 µM ART, whereas a concentration of 30 µM ART was necessary to significantly decrease proliferation in parental A-498 cells (Figure 2d).

### 2.4. Artesunate Reduces Clonogenic Growth of the RCC Cell Lines

**Figure 3:**
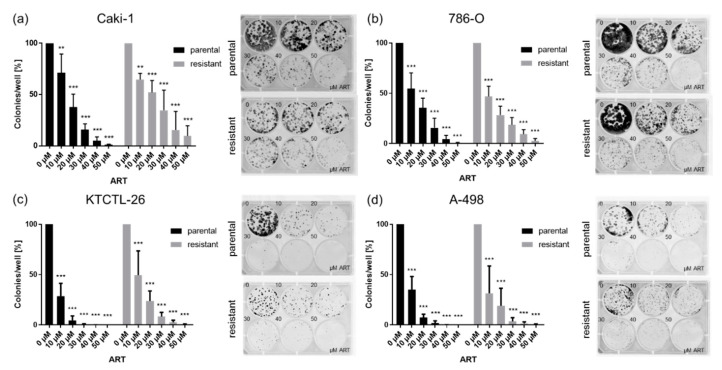
Clonogenic growth of RCC cells: Clonogenic growth of parental and resistant Caki-1 (**a**), 786-O (**b**), KTCTL-26 (**c**), and A-498 (**d**) cells treated with ART (10–50 µM) for 10 days. Untreated cells served as controls (set to 100%). Error bars indicate standard deviation (*SD*). Significant difference to untreated control: ** *p* ≤ 0.01, *** *p* ≤ 0.001. *n* = 5.

In all RCC cell lines, ART induced a significant dose-dependent reduction in clone colonies after 10 days incubation (Figure 3). Ten µM ART contributed to significant inhibition of the clonogenic growth of the RCC cells, compared to the untreated controls. In parental and resistant Caki-1 cells, the administration of 50 µM ART diminished the clonogenic growth by more than 90% (Figure 3a). Microscopically, parental Caki-1 cells formed larger colonies, compared to the sunitinib-resistant Caki-1 cells (Figure 3a). Treatment of 786-O cells with 10 µM ART resulted in an approximately 50% decrease in clone colonies (Figure 3b). 786-O cells exposed to 50 µM ART completely inhibited colony formation in the parental and resulted in only a few colonies in the resistant cell line. In parental and sunitinib-resistant KTCTL-26 and A-498 cells, 10 µM ART significantly diminished the clonogenic growth by more than 50% (Figure 3c,d). KTCTL-26 colonies were no longer formed after exposure to 30 µM ART in parental and exposure to 50 µM ART in resistant cells (Figure 3c). Neither parental nor resistant A-498 colonies were detectable after exposure to 40 and 50 µM ART (Figure 3d). Microscopic comparison showed that both parental and resistant A-498 cells exhibited a lower potential to develop colonies, compared to the other RCC cell lines (Figure 3d).

### 2.5. Artesunate Induces Cell Cycle Arrest in Both Parental and Sunitinib-Resistant RCC Cells

**Figure 4:**
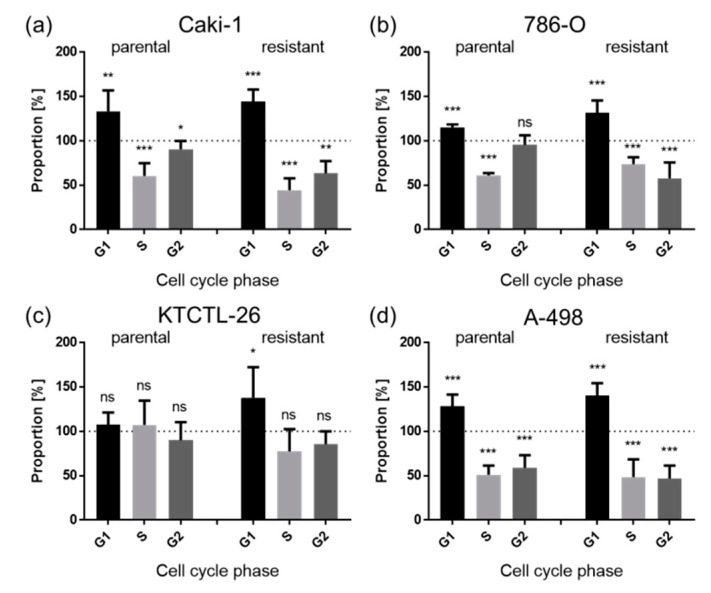
Distribution of cell cycle phases: Proportion of parental and sunitinib-resistant RCC cells, Caki-1 (**a**), 786-O (**b**), KTCTL-26 (**c**), and A-498 (**d**), in the G0/G1, S, and G2/M phases after 48 h treatment with ART (20 µM). Untreated cells served as controls (dotted line; set to 100%). Error bars indicate standard deviation (*SD*). Significant difference to untreated control: * *p* ≤ 0.05, ** *p* ≤ 0.01, *** *p* ≤ 0.001, ns = not significant. *n* = 5.

Diminished growth behavior in the parental and sunitinib-resistant RCC cell lines Caki-1, 786-O, and A-498 was accompanied by a significant G0/G1 phase arrest after exposure to ART, compared to the untreated controls (Figure 4a,b,d). Concomitantly, the number of S and G2/M phase cells significantly decreased, except in the parental 786-O cells, exhibiting only a significant reduction in the S phase (Figure 4b). In KTCTL-26 ART induced a significant G0/G1 phase arrest in the resistant cells, but no changes were apparent in the parental counterpart (Figure 4c). Overall, the effect of ART on KTCTL-26 regarding induction of cell cycle arrest was less pronounced than in the other RCC cell lines. To explore the influence of ART on cell cycle regulating protein levels, Caki-1 and 786-O were utilized as exemplary cell lines.

### 2.6. Artesunate-Induced Cell Cycle Arrest was Accompanied by Alterations in the Expression and Activity of Cell Cycle Regulating Proteins

**Figure 5:**
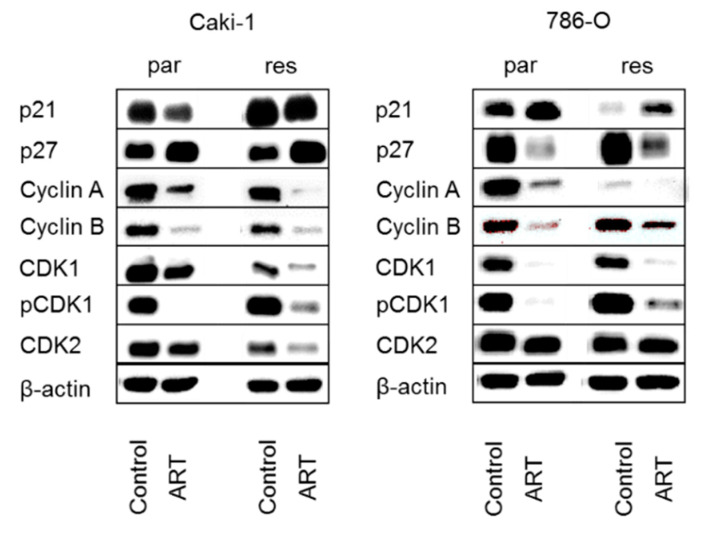
Protein expression profile of cell cycle regulating proteins: Representative Western blot analysis of cell cycle regulating proteins in parental (par) and sunitinib-resistant (res) Caki-1 (**left** panel) and 786-O (**right** panel) cells after 48 h exposure to ART (20 µM).

**Figure 6:**
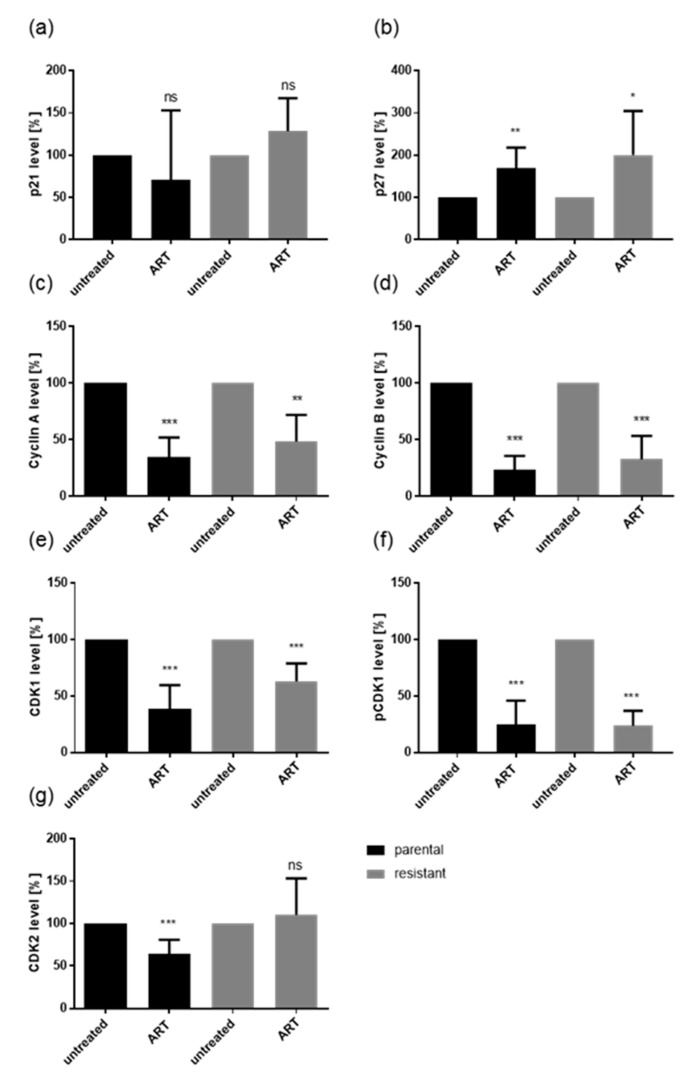
Protein expression profile of cell cycle regulating proteins: Pixel density analysis (Western blot) of the cell cycle regulating proteins p21 (**a**), p27 (**b**), cyclin A (**c**), cyclin B (**d**), CDK1 (**e**), pCDK1 (**f**), and CDK2 (**g**) in parental and resistant Caki-1 cells after 48 h exposure to ART (20 µM), compared to untreated controls (set to 100%). Each protein analysis was accompanied and normalized by a housekeeping protein. Error bars indicate standard deviation (*SD*). Significant difference to untreated control: * *p* ≤ 0.05, ** *p* ≤ 0.01, *** *p* ≤ 0.001, ns = not significant. *n* = 4. For detailed information regarding the Western blots see Figure S1a–g.

**Figure 7:**
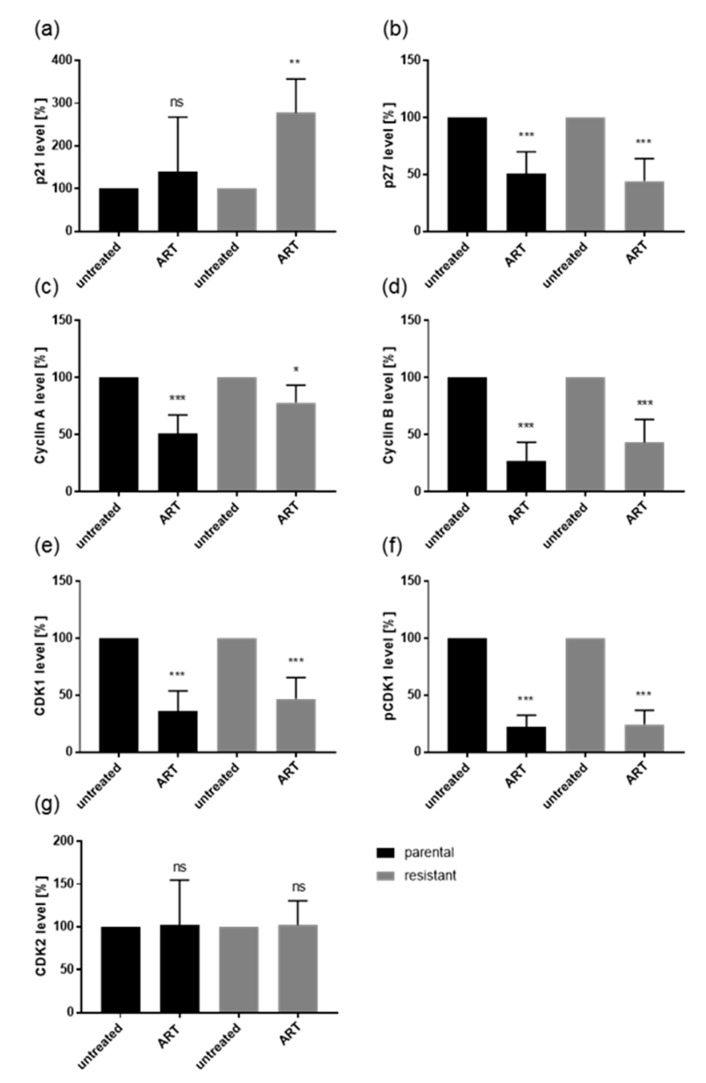
Protein expression profile of cell cycle regulating proteins: Pixel density analysis (Western blot) of the cell cycle regulating proteins p21 (**a**), p27 (**b**), cyclin A (**c**), cyclin B (**d**), CDK1 (**e**), pCDK1 (**f**), and CDK2 (**g**) in parental and resistant 786-O cells after 48 h exposure to ART (20 µM), compared to untreated controls (set to 100%). Each protein analysis was accompanied and normalized by a housekeeping protein. Error bars indicate standard deviation (*SD*). Significant difference to untreated control: * *p* ≤ 0.05, ** *p* ≤ 0.01, *** *p* ≤ 0.001, ns = not significant. *n* = 4. For detailed information regarding the Western blots see Figure S2a–g.

Alterations in the cell cycle phases of Caki-1 and 786-O after administration of ART were associated with distinct modulation in cell cycle regulating proteins (Figure 5, Figure 6, Figure 7). The treatment of parental and sunitinib-resistant Caki-1 and 786-O cells with 20 µM ART led to a significant reduction of the cell cycle activating proteins cyclin A, cyclin B, and CDK1, as well as to deactivation of CDK1 (pCDK1), all of which are proteins involved in S and G2/M phase progression. In addition, CDK2, which associates with cyclin A during the S phase, significantly decreased in parental Caki-1 cells after exposure to ART, compared to the untreated controls (Figure 5 and Figure 6g, Figure S1g). The expression of the tumor suppressor p27 significantly increased in parental and resistant Caki-1 cells with ART application (Figure 5 and Figure 6b, Figure S1b). However, in 786-O cells, p27 expression significantly decreased after ART application (Figure 5 and Figure 7b, Figure S2b). Protein expression of p21 was not significantly altered in Caki-1 cells (Figure 5 and Figure 6a, Figure S1a) but tended to increase in parental 786-O cells and was significantly increased in resistant 786-O cells (Figure 5 and Figure 7a, Figure S2a). Activity of CDK2 (pCDK2) was not detectable in Caki-1 and 786-O cells. 

### 2.7. Artesunate Only Slightly Contributes to Apoptosis

**Figure 8:**
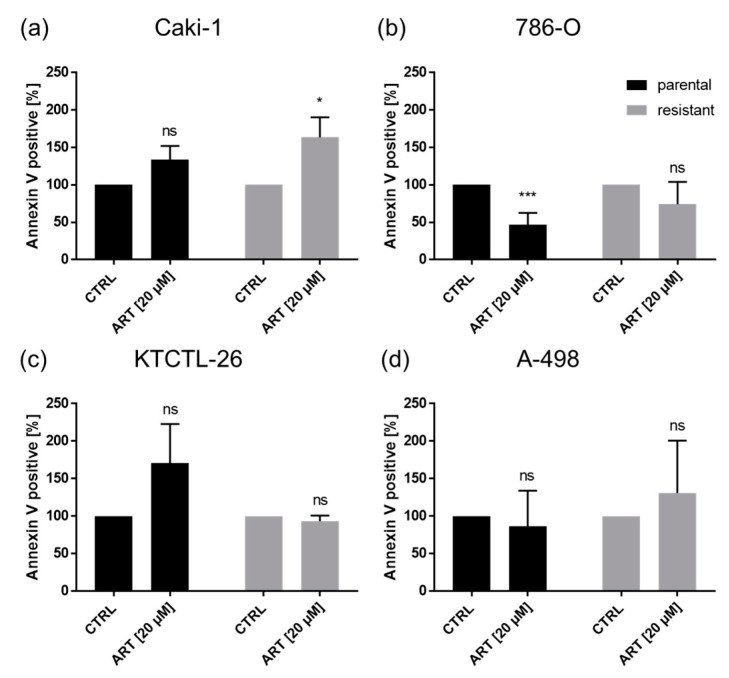
Apoptotic events: Parental and resistant Caki-1 (**a**), 786-O (**b**), KTCTL-26 (**c**), and A-498 (**d**) cells treated for 48 h with ART (20 µM). Untreated cells served as controls (set to 100%). Error bars indicate standard deviation (*SD*). Significant difference to untreated control: * *p* ≤ 0.05, *** *p* ≤ 0.001, ns = not significant. *n* = 5.

To investigate whether the growth inhibitory effect of ART was associated with cell death events, apoptosis was assessed (Figure 8). The only significant increase in apoptotic cells in parental or sunitinib-resistant RCC cells after exposure to ART occurred in resistant Caki-1 cells (Figure 8a).

### 2.8. Artesunate Results in Ferroptosis Induction in KTCTL-26 Cells

**Figure 9:**
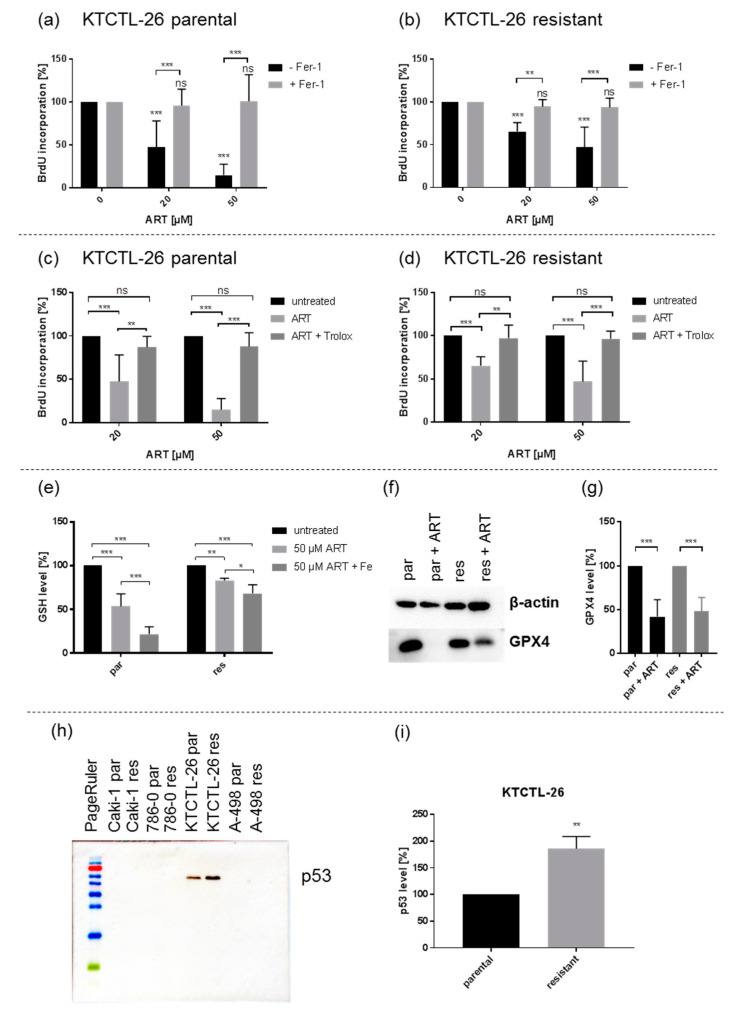
Artesunate induced ferroptosis by reactive oxygen species (ROS) formation in p53-positive KTCTL-26 cells: Ferroptosis induction (**a**,**b**) Proliferation of parental (**a**) and sunitinib-resistant KTCTL-26 cells (**b**) treated for 48 h with ART (20, 50 µM) and ferrostatin-1 (Fer-1) (20 µM). Untreated (100%) and ART mono-treated cells served as controls. Error bars indicate standard deviation (*SD*). Significant difference compared to untreated controls, except for asterisk brackets indicating significant difference between ferrostatin-1 untreated and treated cells: * = *p* ≤ 0.05, ** = *p* ≤ 0.01, *** = *p* ≤ 0.001, ns = not significant. *n* = 5. Indications of ROS generation (**c**–**g**): Proliferation of parental (**c**) and sunitinib-resistant KTCTL-26 cells (**d**) treated for 48 h with ART (20, 50 µM) and Trolox (0.5 mM). Untreated cells served as controls (100%). *n* = 5. GSH level (%) of parental (par) and resistant (res) KTCTL-26 cells after 24 h incubation with ART (50 µM) and holo-transferrin (Fe) (**e**). Untreated controls served as controls (100%). *n* = 5. GPX4 expression: Representative Western blot of GPX4 expression in parental (par) and sunitinib-resistant (res) KTCTL-26 cells after 48 h exposure to ART (50 µM) (**f**). Pixel density analysis of GPX4 level (%) after 48 h exposure to ART (50 µM) in parental (par) and resistant (res) KTCTL-26 cells (**g**). Untreated cells served as controls (100%). β-actin served as internal control. *n* = 5. Protein expression of p53 (**h**,**i**) Representative Western blot analysis of p53 in parental (par) and resistant (res) Caki-1, 786-O, KTCTL-26, and A-498 cells (**h**). Pixel density analysis of p53 expression in parental (100%) and resistant KTCTL-26 cells (**i**) p53 protein analysis was accompanied and normalized by a total protein control. *n* = 3. Error bars indicate standard deviation (*SD*). Significant difference indicated by: * *p* ≤ 0.05, ** *p* ≤ 0.01, *** *p* ≤ 0.001, ns = not significant. For detailed information regarding the Western blots of (**f**) and (**h**) see Figures S3 and S4.

Since ART has been shown to induce the iron-dependent cell death termed ferroptosis [35,36,38], this type of cell death was investigated by utilizing ferrostatin-1, a ferroptosis inhibitor. Proliferation inhibition observed under ART exposure in parental and sunitinib-resistant KTCTL-26 cells was significantly reversed following the combined administration of ART with the ferroptosis inhibitor ferrostatin-1 (Figure 9a,b). This cancellation of ART’s inhibitory effect caused proliferation rates to return to those of the untreated control cells. Application of ferrostatin-1 did not cancel ART’s proliferation inhibition in parental or sunitinib-resistant Caki-1, 786-O, and A-498 cells. Thus, only the KTCTL-26 cell lines were investigated in further detail.

An essential process during ferroptosis is ROS generation. To investigate whether ART in fact generates ROS, Trolox, an antioxidant was used to intercept free radicals and thus prevent ferroptosis. ART in combination with Trolox significantly abrogated the proliferation inhibition observed with ART treatment alone, so that the proliferation rate of the KTCTL-26 cells was comparable to that of the untreated controls (Figure 9c,d). To further corroborate the results of the aforementioned experiments, glutathione (GSH) expression, a part of the anti-oxidative protective system of the cells, was evaluated. GSH significantly decreased in KTCTL-26 cells after treatment with 50 µM ART, compared to the untreated controls (Figure 9e), indicating ROS generation and GSH consumption. The GSH content was more strongly reduced in parental than in resistant KTCTL-26 cells. In both parental and sunitinib-resistant KTCTL-26 cells, the inhibitory effect significantly increased when ART was combined with iron (Figure 9e). Since GPX4 is essential for anti-oxidative protection and designated as a ferroptosis related protein, GPX4 expression was also assessed. Application of 50 µM ART resulted in a significant reduction of GPX4 in both parental and resistant KTCTL-26 cells (Figure 9f,g and Figure S3).

p53 has been described as a ferroptosis indicator [49,50]. Thus, the expression of p53 in parental and sunitinib-resistant RCC cells was investigated. Expression of p53 was not detectable in the parental and sunitinib-resistant Caki-1, 786-O, and A-498 cells (Figure 9h and Figure S4). Notably, in KTCTL-26 cells, the only cells where ART did induce ferroptosis, distinct p53 expression in parental and even stronger p53 expression in the resistant cells was detected (Figure 9h,i and Figure S4).

### 2.9. Artesunate Influences the Metabolism of RCC Cells

**Figure 10:**
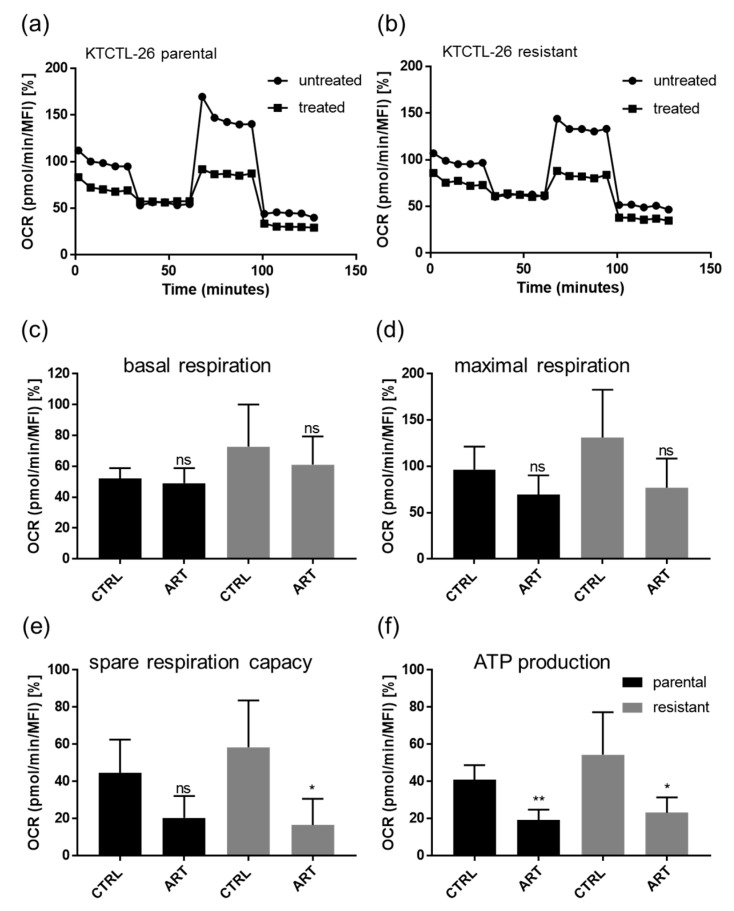
Mitochondrial respiration: Representative mitochondrial respiration in parental (**a**) and resistant (**b**) KTCTL-26 cells after 24 h treatment with 20 µM ART (=treated). Untreated cells served as controls. Data pertaining to the oxygen consumption rate (OCR) were normalized to total basal respiration (set to 100%) consisting of mitochondrial and non-mitochondrial respiration. Extracted values for mitochondrial basal oxygen consumption rate (OCR) (**c**), maximal OCR (**d**), respiratory reserve capacity (**e**), and adenosine triphosphate (ATP) production (**f**) after 24 h ART application (ART). MFI = mean fluorescence intensity. Error bars indicate standard deviation (*SD*). Significant difference to untreated control: * *p* ≤ 0.05, ** *p* ≤ 0.01, ns = not significant. *n* = 4.

The ART-induced ferroptosis in KTCTL-26 cells was accompanied by a significant increase in ROS. Since ferroptosis is associated with a high iron content and accelerated metabolism, ART’s impact on the oxygen consumption rate (OCR) of the KTCTL-26 cells, expressed by basal respiration, adenosine triphosphate (ATP) production-coupled respiration, maximum and reserve capacities, and non-mitochondrial respiration, was assessed (Figure 10). Exposure to ART significantly inhibited the spare respiratory capacity, representing the ability of cells to enhance respiration in response to physiological or pharmacological stress, in resistant KTCTL-26 cells (Figure 10e). Decreased spare respiration capacity in the resistant KTCTL-26 cells was accompanied by diminished ATP production (Figure 10f). Also, in parental RCC cells, ATP production significantly decreased after exposure to ART (Figure 10f). ART exerted no significant effect on basal or maximum respiration in either parental or resistant cells (Figure 10c,d). No alteration in the extracellular acidification rate connected with anaerobic glycolytic activity was observed after exposure to ART in the KTCTL-26 cells, thus indicating no shift towards compensatory glycolysis.

## 3. Discussion

Although current therapeutic approaches have improved progression-free survival of advanced RCC patients, the disease at this stage ultimately remains incurable due to the inevitable development of resistance to treatment. Interestingly, in the current study RCC cells exhibiting a more sensitive initial response to sunitinib developed strong resistance in the course of chronic treatment. Sunitinib-resistant Caki-1 cells were nearly 10-fold less sensitive than their parental counterparts, which initially could be held in check by a relatively low sunitinib dose. Overcoming this resistance is therefore of primary importance. Since adding ART to conventional anti-cancer therapy has been shown to overcome resistance during treatment of other tumor entities, the impact of ART on a panel of sunitinib-sensitive and sunitinib-resistant RCC cell lines was investigated.

Exposure to ART resulted in a significant inhibition of tumor cell growth and proliferation in all tested parental and sunitinib-resistant RCC cells, indicating an anti-tumor potential in highly heterogenic types of cancer. KTCTL-26 cells displayed the highest sensitivity to ART. The IC50 for ART in the RCC cells was in the lower one- to two-digit µM range (2 to 18 µM). In good accordance with other investigators, ART has been shown to inhibit cell growth of therapy-sensitive RCC cells in the two-digit µM range, up to 50 µM [47]. Combined administration of ART with sorafenib, a first-generation TKI akin to sunitinib, even further reduced cell growth [47]. Several studies on non-urological tumor entities have also demonstrated growth inhibition after ART application. In hemangioendothelioma cells, ART time- and dose-dependently reduced tumor cell growth, concomitantly decreasing the expression of VEGF-A, VEGFR1, VEGFR2, and HIF-1α [46]. Thus, it has been postulated that ART may hold promise in treating vascular tumors, of which RCC is a member. Moreover, significant growth inhibition was observed in a mouse model of hemangioendothelioma carcinoma cells following ART treatment, with significantly reduced tumor size [46]. In different gastric cancer cell lines, ART exposure has also resulted in a significant growth reduction [51]. In bone tumor cell lines, ART also impacted tumor cell growth [52]. In the present investigation, the growth of KTCTL-26 cells, a bone tumor cell line, was even diminished to below zero after ART treatment. This was accompanied by an increased number of annexin V positive cells, indicating apoptosis induction by ART. Other investigators have reported an anti-proliferative effect of ART in ovarian carcinoma cells in vitro [53] as well as in chemotherapy-sensitive and -resistant thyroid cancer cells [54]. In patients with colorectal cancer, ART application reduced disease progression through anti-proliferative action [31]. Artemisinin, the native lead compound, also inhibited proliferation in gastric cancer cell lines by up-regulating p53 [55]. Nevertheless, ART inhibits tumor cells by both p53-dependent and also -independent mechanisms [56,57].

Clonogenic growth provides information about the growth of single tumor cells at metastatic sites and advanced RCC is characterized by its ability to spread and survive at these sites. A prolonged 10 µM ART exposure of up to 10 days contributed to a significant reduction of clone colonies in all parental and respective sunitinib-resistant RCC cell lines. KTCTL-26, but also A-498 cells displayed a high sensitivity towards ART with regard to clone colony formation, followed by 786-O and Caki-1. In good accordance with Jeong et al., a significant decrease in clonogenic growth in therapy-sensitive Caki-1 and 786-O cells has been shown [47]. Not only ART, but also artemisinin significantly diminished clonogenic growth in therapy-sensitive RCC cells by down-regulating AKT, a survival protein, and up-regulating E-cadherin, an epithelial differentiation marker [58]. E-cadherin loss is associated with poor prognosis and continued spread of disease [59]. Since AKT up-regulation and loss of E-cadherin have previously been demonstrated in therapy-resistant RCC cells [60,61,62,63,64], it is conceivable that these proteins are also affected by ART in the sunitinib-resistant RCC cells.

Reduced cell growth and proliferation in response to ART were associated with impaired cell cycle progression. Parental and resistant Caki-1, 786-O, and A-498 cells displayed a significant G0/G1 phase arrest after exposure to 20 µM ART. Accumulation of the cells in G0/G1 correlated with a significant reduction of the cells in the S and G2/M phase. However, parental KTCTL-26 cells were not affected, and their sunitinib-resistant counterparts were only moderately affected. Concordant with the present investigation, ART and other derivatives of artemisinin have been shown to promote cell cycle arrest in the G0/G1 phase in several tumor entities [65,66]. Application of ART in epidermoid carcinoma cells has been shown to halt cells in the G0/G1 phase [65]. Artemisinin application has resulted in a similar effect in cell cultures from endometrial tumors [66]. In therapy-sensitive RCC cells, Caki-1, and 786-O, 50 µM ART has been shown to induce a G2/M phase arrest [47], whereas the G0/G1 cell cycle arrest observed in the present investigation was induced with 20 µM ART. These differences in cell cycle arrest could be due to the different ART concentrations. Depending on dose, other investigators have demonstrated a ROS-dependent cell cycle arrest induced by ART in both the G0/G1 and G2/M phases in breast cancer cells [67].

Cell cycle progression is controlled by alternating CDK-cyclin complexes. In good accordance with the G0/G1 cell cycle arrest induced by ART, the cell cycle activating proteins CDK 1/2 and cyclin A/B, responsible for S and G2/M phase progression, were down-regulated, whereas the cell cycle inhibiting proteins, p21 in 786-O cells, and p27 in Caki-1 cells, were elevated. The CDK2-cyclin A complex mediates DNA replication in the S phase [68]. The CDK1-cyclin A complex promotes S phase transition, and CDK1-cyclin B complex drives transition from the G2 to M phase [69]. Hence, there is strong evidence that CDK1, CDK2, cyclin A, and B down-regulation by ART evokes the G0/G1 phase arrest, inhibiting growth of the RCC cells. Indeed, blocking CDK1/2 or cyclin A/B by small interfering RNA has been shown to significantly reduce cell growth in Caki-1, KTCTL-26, and A-498 cells [18,19]. Increased p21 and p27 after ART application are also indicative of cell cycle arrest, as both proteins mediate cell cycle arrest in the G0/G1 phase [70,71]. In epidermoid cancer cells, administration of ART resulted in a G0/G1 phase arrest and concomitant p27 increase [65]. Consistent with the current investigation, cell cycle arrest of the epidermoid cancer cells after ART treatment correlated with down-regulation of cyclin A1, cyclin B, and CDK2 [65]. However, in 786-O cells the expression of p27 was already high and significantly diminished after ART exposure. Studies on bone cancer have demonstrated that p27 in addition to its anti-tumor function can play a role in oncogenesis [72]. In line with this, for some renal cell carcinomas, increased p27 expression was associated with worse prognosis [73]. This may hold true for the 786-O cell lines but remains speculative and requires further investigation. Thus, ART seems to act cell type-dependently, attributable to the initial protein content and/or stage of disease. This might also be clinically important with regard to the intra-tumor heterogeneity of RCC [74], since RCC is a tumor entity harboring varying molecular signatures with different sensitivity to treatment [75].

Evidence has been presented showing that cell death may also be responsible for growth inhibition by ART [47,51]. However, in the current study, only in sunitinib-resistant Caki-1 cells were significant apoptotic effects apparent after ART treatment. Based on the dose–response curves and the fact that KTCTL-26 cells reveal no or just slight effects on cell cycle progression, it might be assumed that ART enables cell death in the KTCTL-26 cell line. However, ART did not induce apoptosis in the KTCTL-26 cells. Furthermore, 786-O and A-498 cell lines also displayed no apoptosis induction under ART treatment. Other investigators have shown that ART induced apoptosis in tumor cells, but often only after application of higher ART concentrations than used in the current study. In stomach tumors, apoptotic events were detected in vitro with concentrations upwards of 50 µM ART [51]. Similarly, induction of apoptosis was apparent after 48 h exposure to 50 µM ART in therapy-sensitive Caki-1 and 786-O cells [47]. Since the tumor cell growth of Caki-1, 786-O, and A-498 cell lines was also not reduced below the initial cell count at seeding, even with the higher 50 µM ART concentration, apoptosis induction by ART can only play a minor role in controlling RCC. This leaves the question open as to how to explain the reduction in the KTCTL-26 cells below that of the initial seeding count after exposure to ART.

One explanation of the magnitude of this ART-induced growth inhibitory effect might be induction of ferroptosis. Indeed, parental and sunitinib-resistant KTCTL-26 demonstrated a significant reversion in ART’s growth inhibitory effect after additional application of the ferroptosis inhibitor ferrostatin-1, indicating that ART does induce ferroptosis. Accordingly, ferroptotic effects have been demonstrated in cell cultures of head and neck cancer after ART treatment [25]. In pancreatic cancer cells, ART also triggered ferroptosis [36], and sorafenib combined with ART induced ferroptosis in liver cancer cells [76].

Caki-1, 786-O, and A-498 cells did not show any response to ferrostatin-1, indicating that ferroptosis does not take place in these cell lines. The ART-induced growth inhibition in these cells must therefore mainly act through cell cycle arrest.

Since ART induced ROS generation during ferroptosis [48], Trolox, a vitamin E derivative and anti-oxidant that neutralizes ROS [32], was applied to investigate whether the inhibitory effect of ART could be canceled. In both parental and sunitinib-resistant KTCTL-26 cells, the inhibitory effect of ART was significantly reversed, showing that ART can act through ROS generation. Glutathione (GSH), a key regulator of excessive ROS levels [77], was also significantly reduced after ART administration. ART evoked a stronger GSH reduction in parental KTCTL-26 cells than in resistant cells, which might mean that these resistant RCC cells have a higher basal ROS tolerance. Support for this thesis is provided by a proteomic study, showing that glutathione metabolism in sunitinib-resistant 786-O RCC cells was increased 4–5 times compared to parental cells [78]. Hence, adding ART to sunitinib treatment might counteract ROS tolerance in therapy-resistant cells, and facilitate ferroptosis. In the current investigation, combining ART with iron further potentiated the decrease of GSH in both parental and sunitinib-resistant KTCTL-26 cell lines. ART in combination with lysosomal iron led to the development of ROS and ultimately induces apoptosis via the intrinsic pathway [32]. Increased efficacy of ART in the presence of iron has been shown in pancreatic [36] and in breast cancer cell lines [32]. A high iron content within the tumor cells therefore seems to augment ART’s efficacy, and tumor cells with increased iron metabolism could be selectively targeted, including RCC [79].

Phospholipid-hydroxy peroxide-glutathione peroxidase (PHGPx, gene: GPX4) is another key protein involved in augmented ROS generation. Substances containing an endoperoxid group, such as ART, directly inhibit GPX4, first sensitizing “GPX4 tumors” to ferroptosis [80] and ultimately leading to ferroptosis [81]. GPX4 was significantly reduced in parental and resistant KTCTL-26 cells after ART treatment. Over-expression of GPX4 prevented ferroptosis in colorectal cancer in in vitro studies [82]. Consequently, this might also be the case for the KTCTL-26 cell lines.

p53 has been described as a possible ferroptosis enhancer [49,50]. Interestingly, p53 was exclusively expressed in the parental and sunitinib-resistant KTCTL-26 cell lines, the only RCC cell lines demonstrating ferroptosis induction after exposure to ART. p53 inhibited cysteine influx and thus disrupted GSH metabolism [83]. Furthermore, and consistent with our results, ferroptosis could not be induced in p53-defective cells. Hence, p53 expression may impact GSH metabolism and might be a predictor for ferroptosis induction in parental and sunitinib-resistant RCC cells. Still, this is speculative and requires further investigation. In tumor cells inducing apoptosis, cell death is regulated by both p53-dependent and -independent pathways [56,57].

Along with induction of ferroptosis, ART exposure resulted in significantly diminished ATP production and spare reserve capacity in both parental and sunitinib-resistant KTCTL-26 cells, throttling the energy supply necessary for tumor cell progression. Consistent with this, ART administration in B-cell lymphoma cells and prolactinoma cells led to reduced ATP production [84,85].

The results presented here show that ART induced significant growth inhibitory effects in parental and, more importantly, sunitinib-resistant RCC cells. Although all four RCC cell lines responded to ART, cell type-specific responses were evident. This might give an insight on how ART may act in heterogeneous tumors. In parental and resistant Caki-1, 786-O, and A-498 cells, growth inhibitory effects were accompanied by cell cycle arrest in the G0/G1 phase and respective modulation of the cell cycle regulating proteins. It may therefore be assumed that ART led to growth and proliferation inhibition, but not to tumor cell death. In contrast, parental and sunitinib-resistant KTCTL-26 cells were mainly affected by ART through ferroptosis and decreased metabolism, leading to both growth inhibition and tumor cell death. Notably, p53 was only evident in the KTCTL-26 cells, indicating that p53 might be predicative for ART-dependent ferroptosis and induce a more effective drug response, which could be clinically relevant. The in vitro data give a first insight into the anti-tumor activity of ART in RCC cells that might in its strength and the respective mechanism depend on the initial protein profile of the tumor cells and therewith aspects of the intra-tumor heterogeneity. However, since in vitro data reveal an isolated tumor cell system, further investigations are necessary to verify our postulates and in vivo studies need to clarify whether ART shows similar anti-tumor effects under physical conditions in parental and sunitinib-resistant RCC.

## 4. Materials and Methods

### 4.1. Cell Cultures

Renal cell carcinoma cell lines Caki-1, 786-O, KTCTL-26, and A-498 were kindly provided by Prof. Dr. Roman Blaheta (Department of Urology, University Hospital Frankfurt, Goethe-University, GER), initially purchased from Promocell (LGC Promochem, Wesel, Germany). Caki-1 cells were grown and sub-cultured in Iscove Basal medium (Biochrom GmbH, Berlin, Germany), 786-O, KTCTL-26, and A-498 were grown in RPMI-1640 medium (Gibco, Thermo Fisher Scientific, Darmstadt, Germany). Media were supplemented with 10% fetal calf serum (FCS) (Gibco, Thermo Fisher Scientific, Darmstadt, Germany), 1% glutamax (Gibco, Thermo Fisher Scientific, Darmstadt, Germany), and 1% Anti/Anti (Gibco, Thermo Fisher Scientific, Darmstadt, Germany). Twenty mM HEPES-buffer (Sigma-Aldrich, Darmstadt, Germany) was added to the RPMI-1640 medium. Tumor cells were cultivated in a humidified, 5% CO_2_ incubator. 

### 4.2. Resistance Induction and Application of Sunitinib and Artesunate

Resistance to sunitinib was induced by chronic exposure to ascending sunitinib (free Base, Massachusetts LC Laboratories, Woburn, MA, USA) concentrations from 0.1–1 µM until the cells survived and adapted to the highest dosage. Sunitinib resistance in the RCC cells occurred in average 10 weeks after starting application. Thereafter, they were maintained with 1 µM sunitinib applied three times a week. The IC50 (half-maximal inhibitory concentration) of sunitinib was investigated to verify drug resistance. After starving chronically sunitinib-treated RCC cells for 3 days 0.1–100 µM sunitinib was applied for 72 h. Therapy-sensitive (parental) RCC subcell lines served as controls. RCC cells were designated as sunitinib-resistant when the IC50 under 72 h sunitinib application was approximately doubled.

Artesunate (ART) (Sigma-Aldrich, Darmstadt, Germany) was applied at a concentration of 1–100 μM. Controls (parental and sunitinib-resistant) remained ART-untreated. The IC50 of ART in parental and sunitinib-resistant RCC cells was evaluated analog to sunitinib using the 72 h growth data at a concentration of 1–100 µM ART. To evaluate possible toxic effects of sunitinib and/or ART, cell viability was determined in parallel to experimentation by testing aliquoted cells with trypan blue (Sigma-Aldrich, Darmstadt, Germany). Only viable cells were employed.

### 4.3. Tumor Cell Growth

Cell growth was assessed using 3-(4,5-dimethylthiazol- 2-yl)-2,5-diphenyltetrazolium bromide (MTT) dye. RCC cells (50 µL, 1 × 10^5^ cells/mL) were seeded onto 96-well-plates. After 24, 48, and 72 h, 10 µL MTT (0.5 mg/mL) (Sigma-Aldrich, Darmstadt, Germany) was added for 4 h. Cells were then lysed in 100 µL solubilization buffer containing 10% SDS in 0.01 M HCl. The 96-well-plates were subsequently incubated overnight at 37 °C, 5% CO_2_. Absorbance at 570 nm was determined for each well using a multi-mode microplate-reader (Tecan, Spark 10 M, Crailsheim, Germany). After subtracting background absorbance and offsetting with a standard curve, results were expressed as mean cell number. To illustrate dose-response kinetics, mean cell number after 24 h incubation was set to 100%. Each experiment was done in triplicate.

### 4.4. Proliferation

Cell proliferation was measured using a BrdU (Bromodeoxyuridine / 5-bromo-2′-deoxyuridine) cell proliferation enzyme-linked immunosorbent assay (ELISA) kit (Calbiochem/Merck Biosciences, Darmstadt, Germany). Tumor cells (50 µL, 1 × 10^5^ cells/mL), seeded onto 96-well-plates, were incubated with 20 µL BrdU-labeling solution per well for 24 h, fixed and stained using anti-BrdU mAb according to the manufacturer’s protocol. Absorbance was measured at 450 nm using a multi-mode microplate-reader (Tecan, Spark 10 M, Crailsheim, Germany). Values were presented as percentage compared to untreated controls set to 100%.

### 4.5. Clonogenic Assay

The clonogenic recovery potential gives insight into the capability of the cells to form a new tumor (metastasis). Therefore, 500 cells/well were seeded on a 6-well-plate and treated for 10 days with ART. Untreated cells served as controls. RCC cells were subsequently fixed with 85% MeOH/15% AcOH and stained with Coomassie (0.5 g Coomassie Blue G250 (Sigma-Aldrich, Darmstadt, Germany), 75 mL AcOH, 200 mL MeOH, 725 mL distilled water). Amount and size of cell clone colonies were measured with a biomolecular imager (Sapphire, Azure Biosystems, Biozym, Hess. Oldendorf, Germany). Colony forming efficiency was evaluated by ImageJ analysis. A colony was defined as consisting of at least 50 cells with an area of 50.8 µm^2^. Untreated controls were set to 100%. 

### 4.6. Cell Cycle Phase Distribution

For cell cycle analysis cell cultures were grown to sub-confluency. A total of 1 × 10^6^ cells was stained with propidium iodide (50 µg/mL) (Invitrogen, Thermo Fisher Scientific, Darmstadt, Germany) and then subjected to flow cytometry (Fortessa X20, BD Biosciences, Heidelberg, Germany). Ten thousand events were collected from each sample. Data acquisition was carried out using DIVA software (BD Biosciences, Heidelberg, Germany), and cell cycle distribution was analyzed by ModFit LT 5.0 software (Verity Software House, Topsham, ME, USA). The number of cells in the G0/G1, S, or G2/M phases was expressed as a percentage.

### 4.7. Western Blot Analysis of Cell Cycle Regulating Proteins, GPX4 and p53

To explore the expression and activity of cell cycle and cell death regulating proteins, western blot analysis was performed. Tumor cell lysates (50 µg) were applied to 10% or 12% polyacrylamide gel and separated for 10 min at 80 V and 1 h at 120 V. The protein was then transferred to nitrocellulose membranes (1 h, 100 V). After blocking with 10% non-fat dry milk for 1 h, the membranes were incubated overnight with the following primary antibodies directed against cell cycle proteins: p21 (Rabbit IgG, clone 12D1, Cell Signaling, Frankfurt am Main, Germany), p27 (Mouse IgG_1_, clone 57/Kip1, BD Biosciences, Heidelberg, Germany), Cyclin A (Mouse IgG_1_, clone 25, BD Biosciences, Heidelberg, Germany), Cyclin B (Mouse IgG_1,_ clone 18, BD Biosciences, Heidelberg, Germany), CDK1 (Mouse IgG_1_, clone 2, BD Biosciences, Heidelberg, Germany), pCDK1 (Rabbit, clone 10A11, Cell Signaling, Frankfurt am Main, Germany), CDK2 (Mouse IgG_2a_, clone 55, BD Biosciences, Heidelberg, Germany). 

To indicate lipid peroxidation and ferroptosis related proteins the following primary antibodies were used: GPX4 (Rabbit IgG, ab41787, Abcam, Berlin, Germany), p53 (Rabbit, clone 7F5, Cell Signaling, Frankfurt am Main, Germany). HRP-conjugated rabbit-anti-mouse IgG or goat-anti-rabbit IgG served as secondary antibodies (IgG, both: dilution 1:1000, Dako, Glosturp, Denmark). The membranes were incubated with ECL detection reagent (AC2204, Azure Biosystems, Munich, Germany) to visualize proteins with a Sapphire Imager (Azure Biosystems, Munich, Germany). β-actin (clone AC-1; Sigma Aldrich, Taufenkirchen, Germany) served as the internal control, except for p53, which was normalized to total protein. To quantify total protein all membranes were stained by Coomassie brilliant blue and measured by Sapphire Imager. AlphaView software (ProteinSimple, San Jose, CA, USA) was used for pixel density analysis of the protein bands. The ratio of protein intensity/β-actin intensity or whole protein intensity was calculated and expressed in percentage, related to the untreated controls, set to 100%.

### 4.8. Apoptosis and Ferroptosis

To investigate apoptotic and necrotic events, the FITC-Annexin V Apoptosis Detection kit (BD Biosciences, Heidelberg, Germany) was used to quantify binding of Annexin V/propidium iodide (PI). After washing tumor cells twice with PBS, 1 × 10^5^ cells were suspended in 500 µL of 1 × binding buffer and incubated with 5 µL Annexin V-FITC and (or) 5 µL PI in the dark for 15 min. Staining was measured by flow cytometer (Fortessa X20, BD Biosciences, Heidelberg, Germany). Ten thousand events were collected from each sample. The percentage of apoptotic and necrotic cells in each quadrant was calculated using DIVA software (BD Biosciences, Heidelberg, Germany). Further analysis was done by FlowJo software (BD Biosciences, Heidelberg, Germany). 

A BrdU cell proliferation enzyme-linked immunosorbent assay (ELISA) kit (Calbiochem/Merck Biosciences, Darmstadt, Germany) was used to evaluate ferroptosis and ROS generation. To evaluate ferroptosis, tumor cells were treated for 48 h with 20, 50, and 100 µM ART or ART combined with 20 µM ferrostatin-1 (Sigma-Aldrich, Darmstadt, Germany), a ferroptosis inhibitor. ROS generation during ferroptosis was verified by treating the RCC cells for 48 h with 20, 50, and 100 µM ART in combination with 0.5 mM Trolox (Sigma-Aldrich, Taufkirchen, Germany), an antioxidant. For more details see “Proliferation” (4.4), as described above.

### 4.9. GSH-Assay

The GSH level was evaluated with the GSH-Glo™ Glutathione Assay (Promega Corporation, Madison, Wisconsin, USA). Five thousand cells/well were seeded onto a 96-well-plate and incubated for 24 h with 50 µM ART or ART combined with 20 µg/mL holo-Transferrin (Fe; Sigma-Aldrich, Taufkirchen, Germany). Experiments were performed according to the manufacturer’s protocol. Luminescence was measured using a multi-mode microplate-reader (Tecan, Spark 10 M, Tecan, Grödig, Austria).

### 4.10. Evaluation of Mitochondrial Respiration and Anaerobic Glycolytic Activity

Mitochondrial respiration (OCR = oxygen consumption rate) and anaerobic glycolytic activity (EACR = extracellular acidification rate) were assessed in real time by the Seahorse XFp Extracellular Flux Analyzer using the Seahorse XF Cell Mito Stress Test Kit (both: Agilent Technologies, Waldbronn, Germany). The EACR indicating anaerobic glycolytic activity was used to determine compensatory glycolysis. OCR was obtained by multiple parameters, including basal respiration, ATP production-coupled respiration, maximal and reserve capacities, and non-mitochondrial respiration. Cells stained with CellTracker Green CMFDA (Thermo Fisher Scientific, Darmstadt, Germany) were plated at a density of 2 × 10^4^ cells/well and media was replaced with XF Assay media the following day 1h prior to the assay and incubated without CO_2_. Five measurements of OCR and ECAR were taken at baseline and after each injection of the following mitochondrial modulators: Oligomycin (1.5 µM, Inhibitor of ATP synthase), carbonylcyanide 4-(trifluoromethoxy)phenylhydrazone (FCCP) (1 µM, proton gradient uncoupler), and rotenone/actinomycin A (0.5 µM, inhibitors of complex I/Complex III). Data were normalized by using Wave 2.6.1 (Agilent Technologies, Waldbronn, Germany) desktop software to the mean fluorescent intensity of cells in the area of measurement in each well. Data pertaining to the OCR were normalized to total basal respiration (set to 100%) consisting of mitochondrial and non-mitochondrial respiration. Basal and maximal respiration were calculated by subtracting non-mitochondrial OCR. Respiratory reserve capacity was calculated as the difference between maximal and basal OCR. ATP-linked OCR was estimated as the difference between basal and rotenone/actinomycin A inhibited OCR.

### 4.11. Statistical Analysis

All experiments were performed at least three times. The evaluation and generation of mean values, the associated standard deviation, and normalization in percent were done by Microsoft Excel. Statistical significance was calculated with GraphPad Prism 7.0 (GraphPad Software Inc., San Diego, CA, USA): Two-sided T-test (Western blot, apoptosis, cell cycle), one-way ANOVA test (BrdU), and two-way ANOVA test (MTT). Correction for multiple comparisons was done using the conservative Bonferroni method. Differences were considered statistically significant at a *p*-value ≤ 0.05. 

## 5. Conclusions

ART induced cell-type specific anti-tumor effects in both parental and sunitinib-resistant RCC cells. In three of the four tested cell lines, Caki-1, 786-O, and A-498, ART induced a strong G0/G1 phase arrest. In the KTCTL-26 cell line, the phase arrest was not as pronounced, but ART exposure additionally induced ferroptosis. In this cell line, the anti-tumor activity of ART was much stronger than in the other three cell lines where ferroptosis was not induced by ART. p53 was only detectable in the KTCTL-26 cells, possibly making it a predictive marker for ferroptosis and a better response to ART. Since RCC exhibits intra-tumor heterogeneity, this might be a clinically relevant aspect. The results presented here suggest that ART may hold promise as a new additive therapy option for selected patients with advanced and even sunitinib-resistant RCC.

## Figures and Tables

**Table 1:** Verification of sunitinib resistance: IC50 values of parental and sunitinib-resistant renal cell carcinoma (RCC) cells following 72 h application of 0.1–100 µM sunitinib *n* = 5.

Cell Line	Parental	Resistant	Unit
Caki-1	2.58	19.13	µM
786-O	3.97	11.16	µM
KTCTL-26	6.37	13.31	µM
A-498	10.43	19.30	µM

## Supplementary Materials

The following are available online at https://www.mdpi.com/2072-6694/12/11/3150/s1, Figure S1: Detailed information about Figure 6—Protein expression profile of cell cycle regulating proteins in parental and resistant Caki-1, Figure S2: Detailed information about Figure 7—Protein expression profile of cell cycle regulating proteins in parental and resistant 786-O, Figure S3: Detailed information about Figure 9f—GPX4 expression in parental and sunitinib-resistant KTCTL-26, Figure S4: Detailed information about Figure 9h—Protein expression of p53 in parental and resistant Caki-1, 786-O, KTCTL-26, and A-498 cells.
