# Supplementary material for: Artesunate Inhibits Growth of Sunitinib-Resistant Renal Cell Carcinoma Cells through Cell Cycle Arrest and Induction of Ferroptosis
Source: Cancers (Basel). 2020 Oct 27;12(11):3150. doi: 10.3390/cancers12113150 (PMC7692972; doi:10.3390/cancers12113150)
Supplement: Supplementary file 1 [file cancers-12-03150-s001.pdf]

Supplementary Materials

Artesunate Inhibits Growth of Sunitinib-Resistant Renal Cell Carcinoma Cells through Cell Cycle Arrest and Induction of Ferroptosis

Sascha D. Markowitsch, Patricia Schupp, Julia Lauckner, Olesya Vakhrusheva, Kimberly S. Slade, René Mager, Thomas Efferth, Axel Haferkamp and Eva Juengel

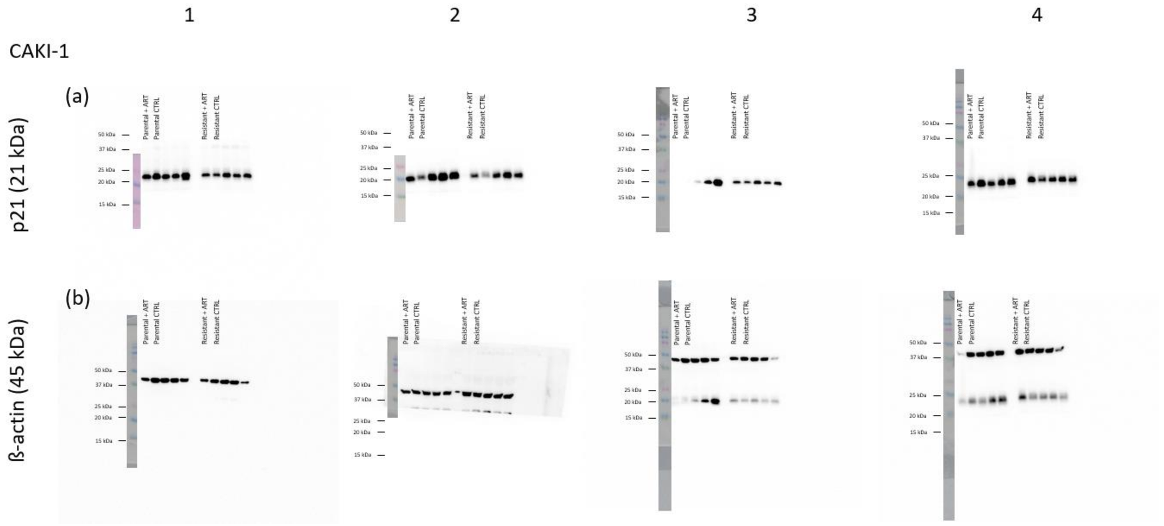

(a)

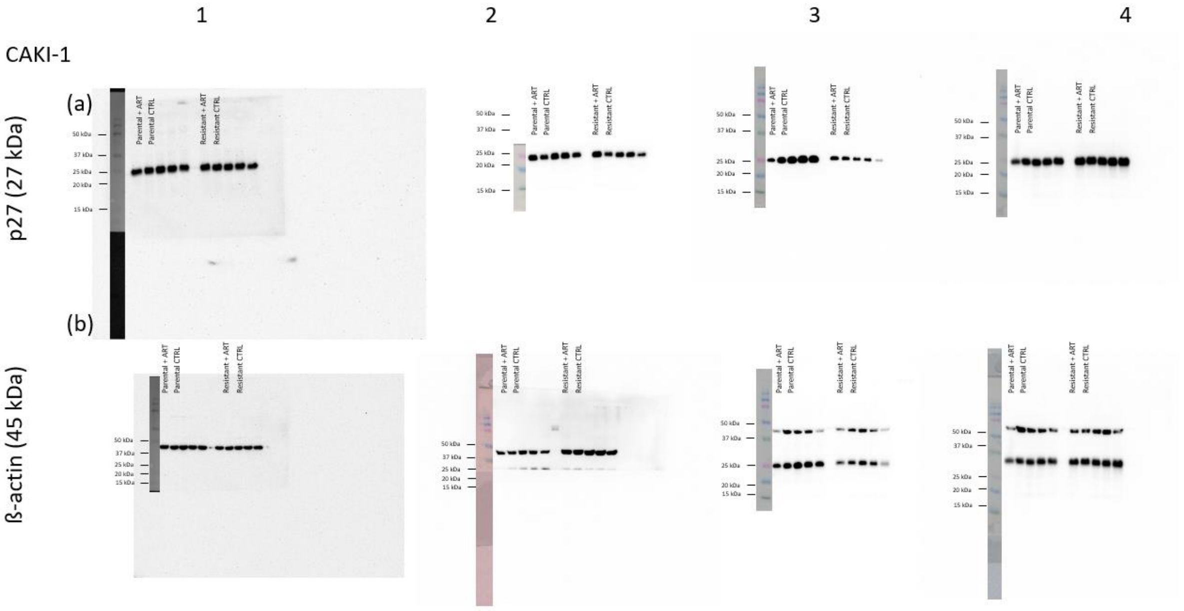

(b)

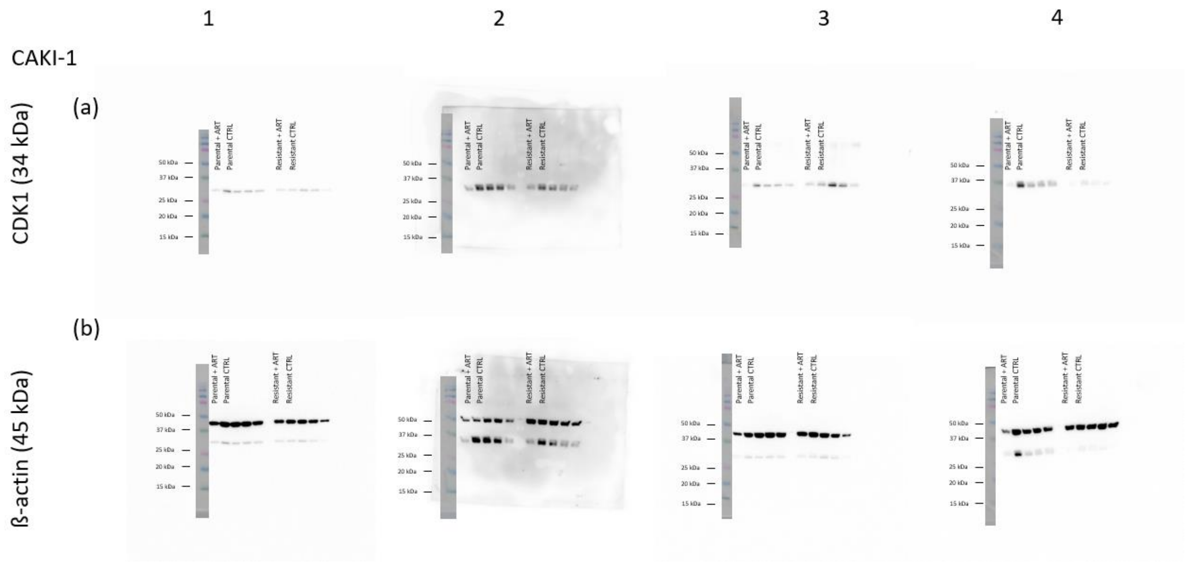
1
2
3
4
CAKI-1
(a)
CDK1 (34 kDa)
(b)
ß-actin (45 kDa)
Parental + ART
Parental CTRL
Resistant + ART
Resistant CTRL

(c)

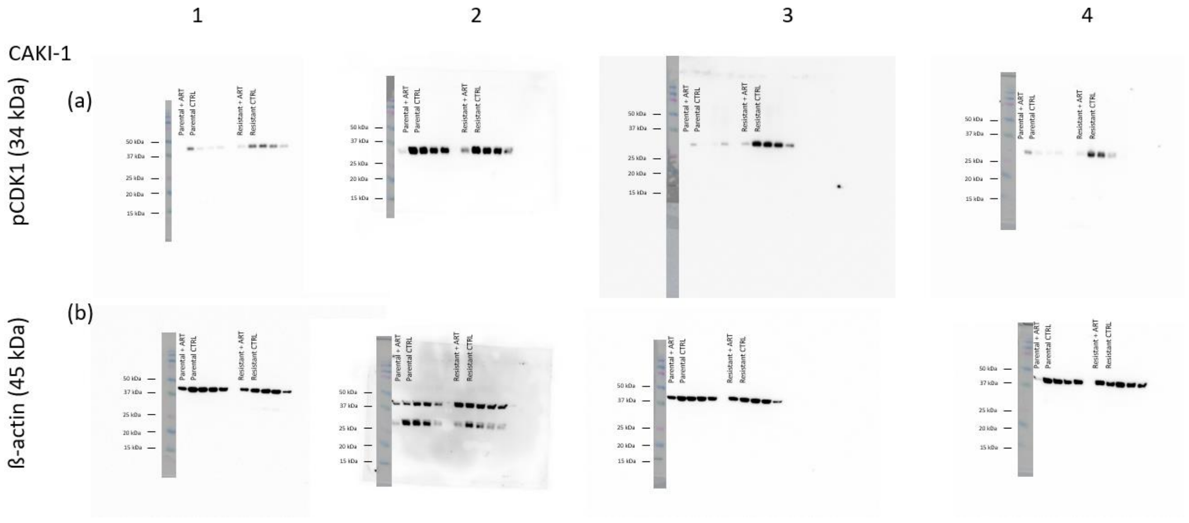
1
2
3
4
CAKI-1
(a)
pCDK1 (34 kDa)
(b)
ß-actin (45 kDa)
Parental + ART
Parental CTRL
Resistant + ART
Resistant CTRL

(d)

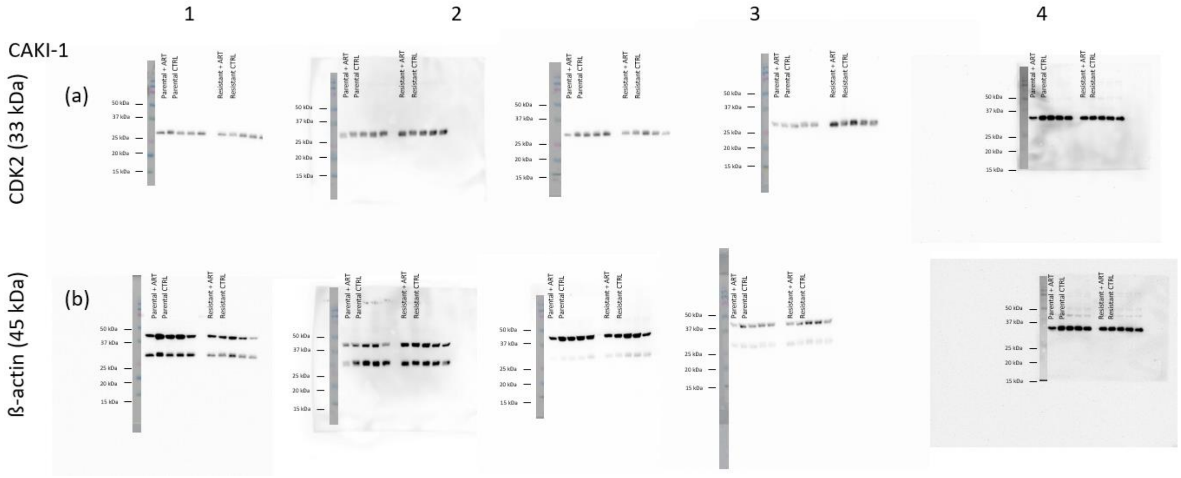
1
2
3
4
CAKI-1
(a)
CDK2 (33 kDa)
(b)
ß-actin (45 kDa)
Parental + ART
Parental CTRL
Resistant + ART
Resistant CTRL

(e)

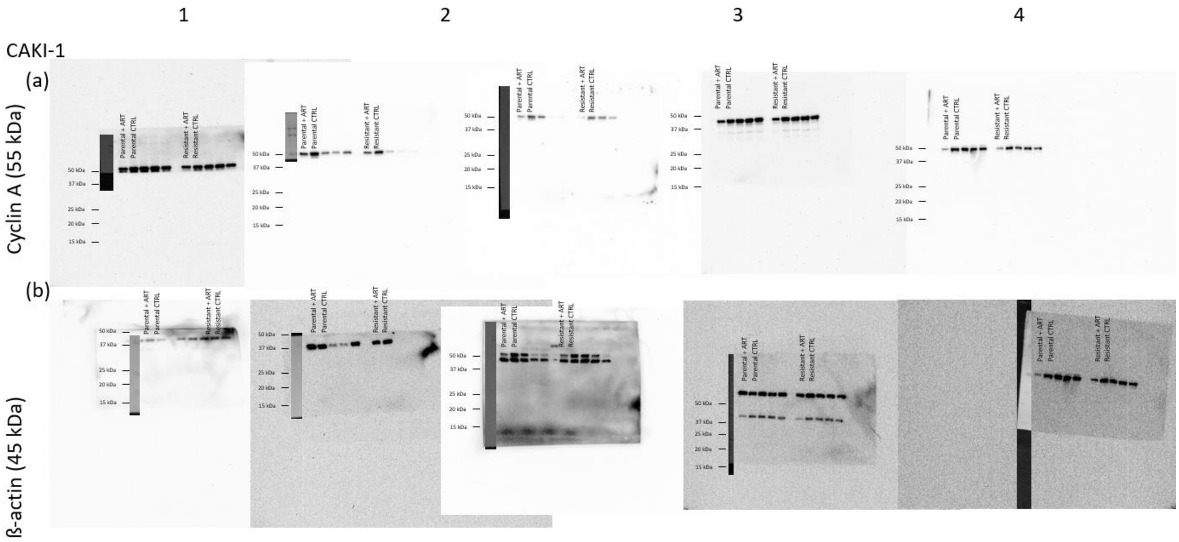

(f)

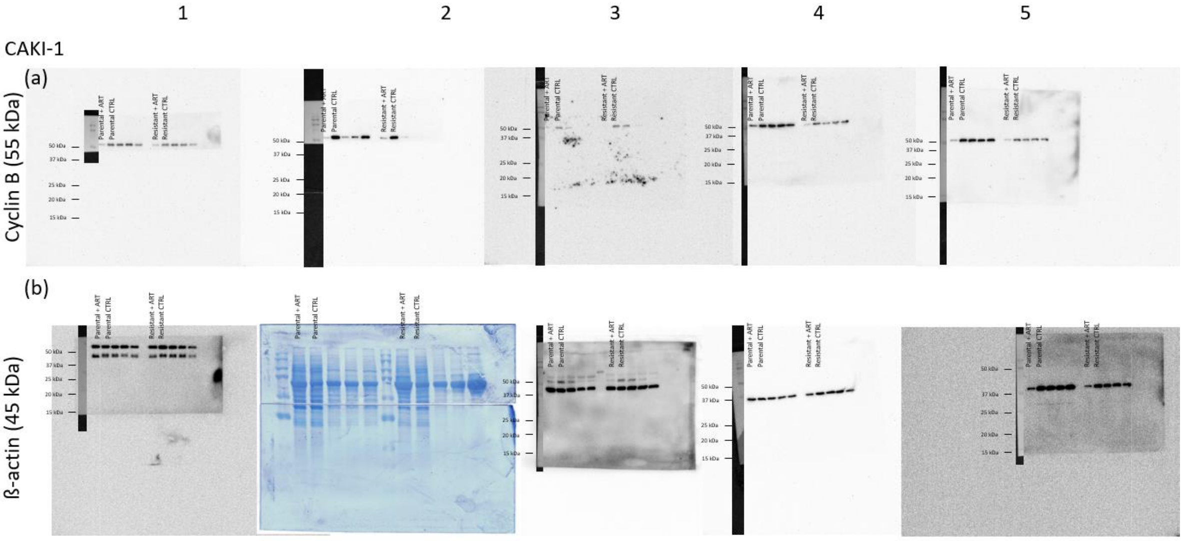

(g)

Figure S1. Detailed information about Figure 6 - Protein expression profile of cell cycle regulating proteins in parental and resistant Caki-1 cells.

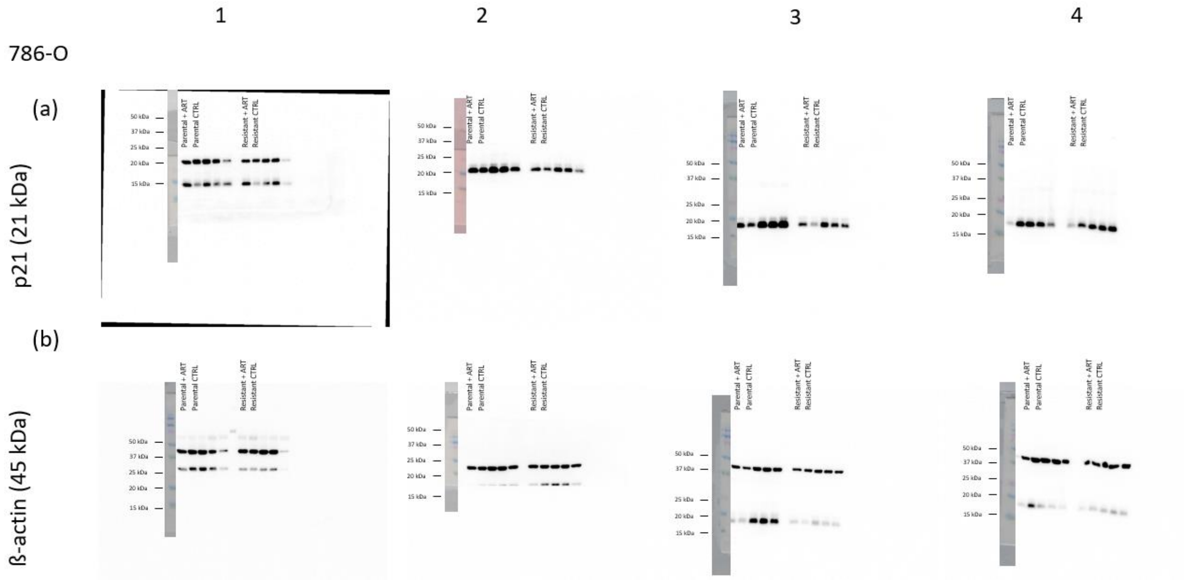
1
2
3
4
786-O
(a)
p21 (21 kDa)
(b)
ß-actin (45 kDa)
Parental + ART
Parental CTRL
Resistant + ART
Resistant CTRL

(a)

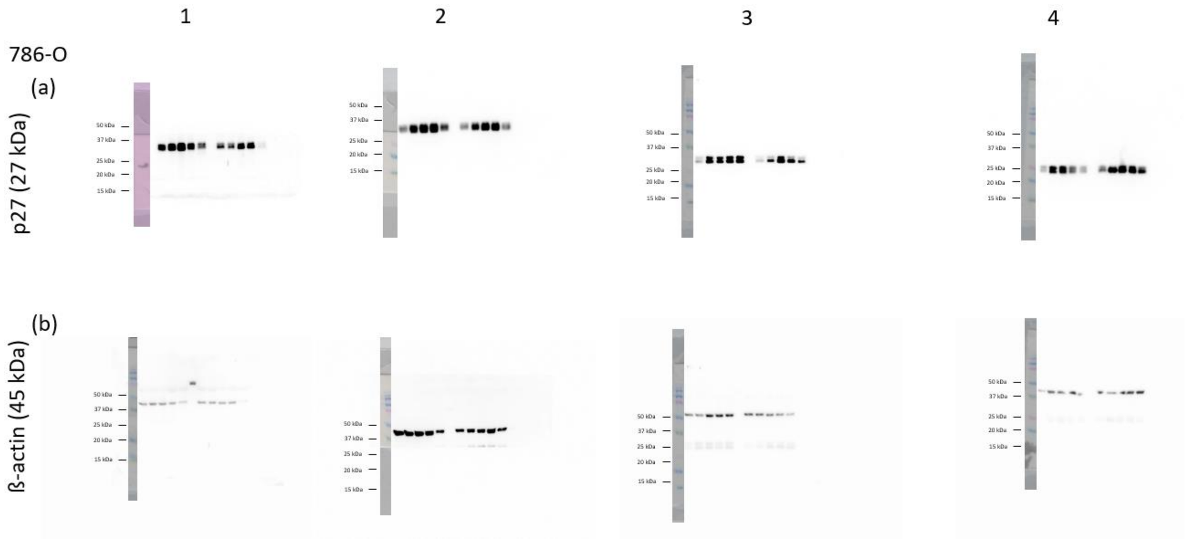
1
2
3
4
786-O
(a)
p27 (27 kDa)
(b)
ß-actin (45 kDa)

(b)

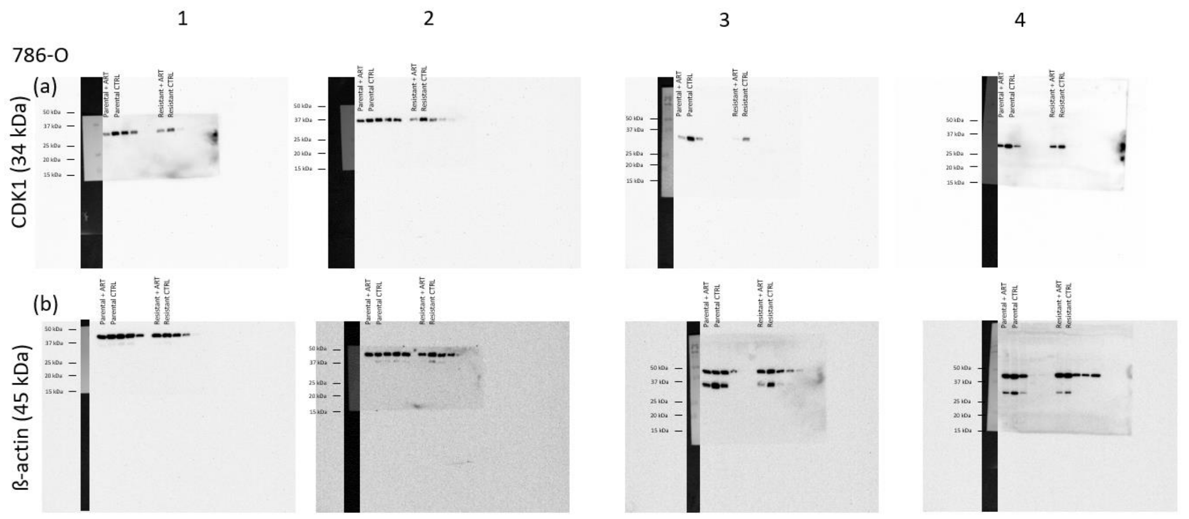
1
2
3
4
786-O
(a)
CDK1 (34 kDa)
(b)
ß-actin (45 kDa)
Parental + ART
Parental CTRL
Resistant + ART
Resistant CTRL

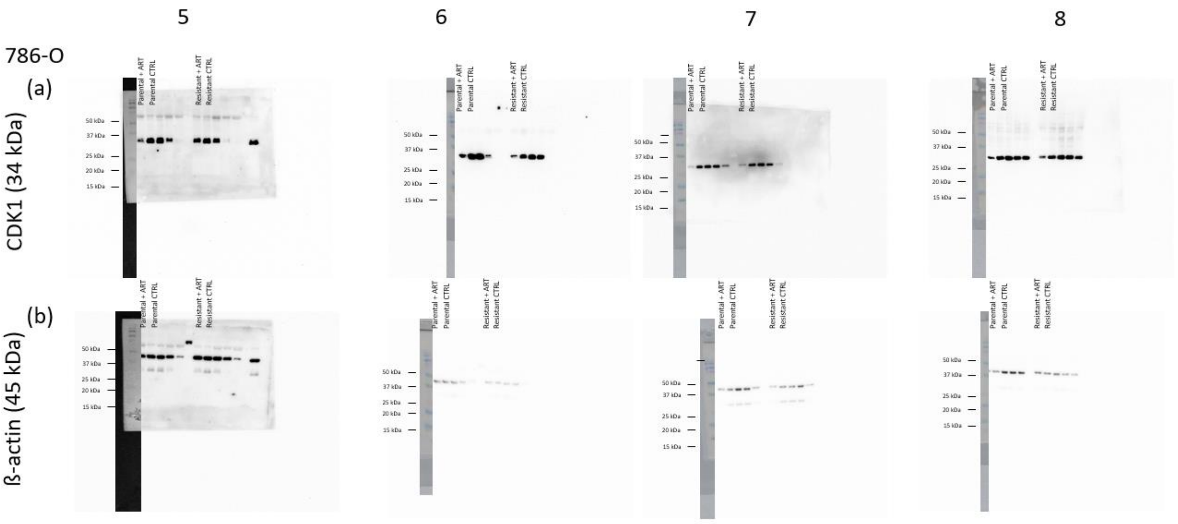
5
6
7
8
786-O
(a)
CDK1 (34 kDa)
(b)
ß-actin (45 kDa)
Parental + ART
Parental CTRL
Resistant + ART
Resistant CTRL

(c)

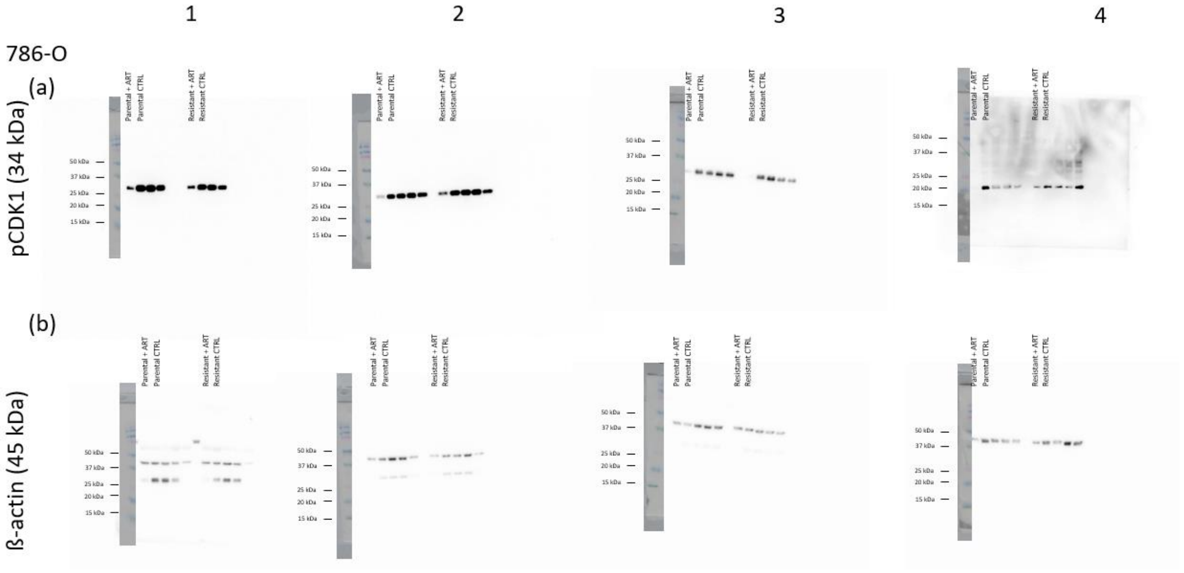
1
2
3
4
786-O
(a)
pCDK1 (34 kDa)
(b)
ß-actin (45 kDa)
Parental + ART
Parental CTRL
Resistant + ART
Resistant CTRL

(d)

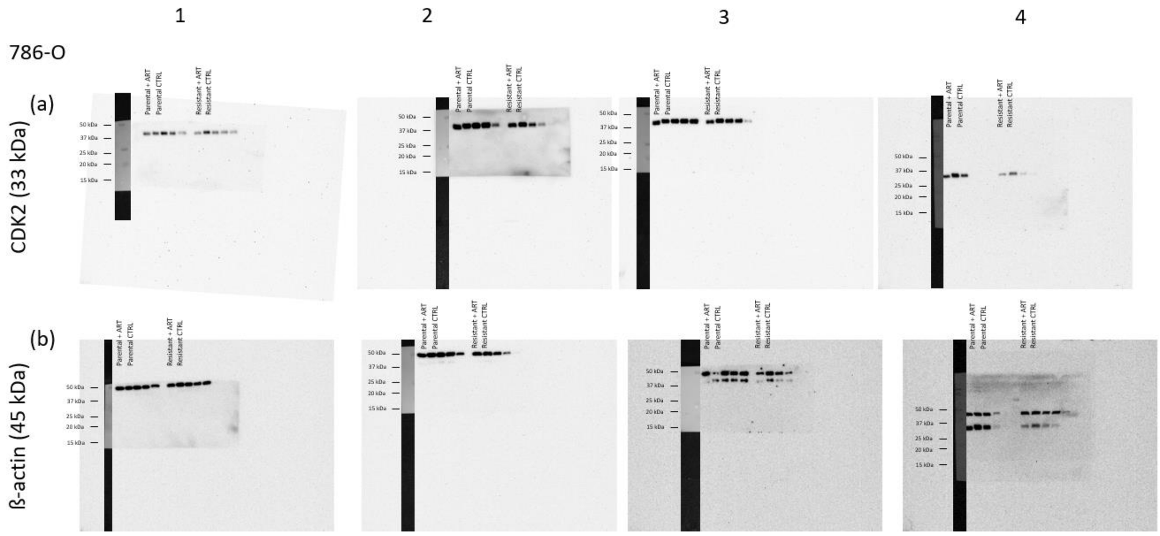
1
2
3
4
786-O
(a)
CDK2 (33 kDa)
(b)
ß-actin (45 kDa)
Parental + ART
Parental CTRL
Resistant + ART
Resistant CTRL

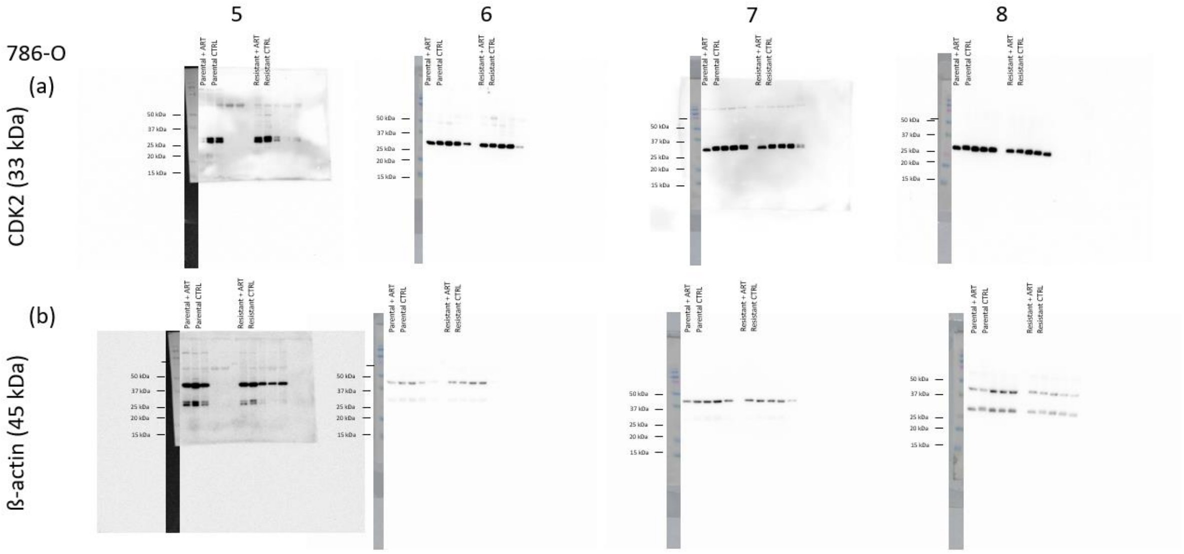
5
6
7
8
786-O
(a)
CDK2 (33 kDa)
(b)
ß-actin (45 kDa)
Parental + ART
Parental CTRL
Resistant + ART
Resistant CTRL

(e)

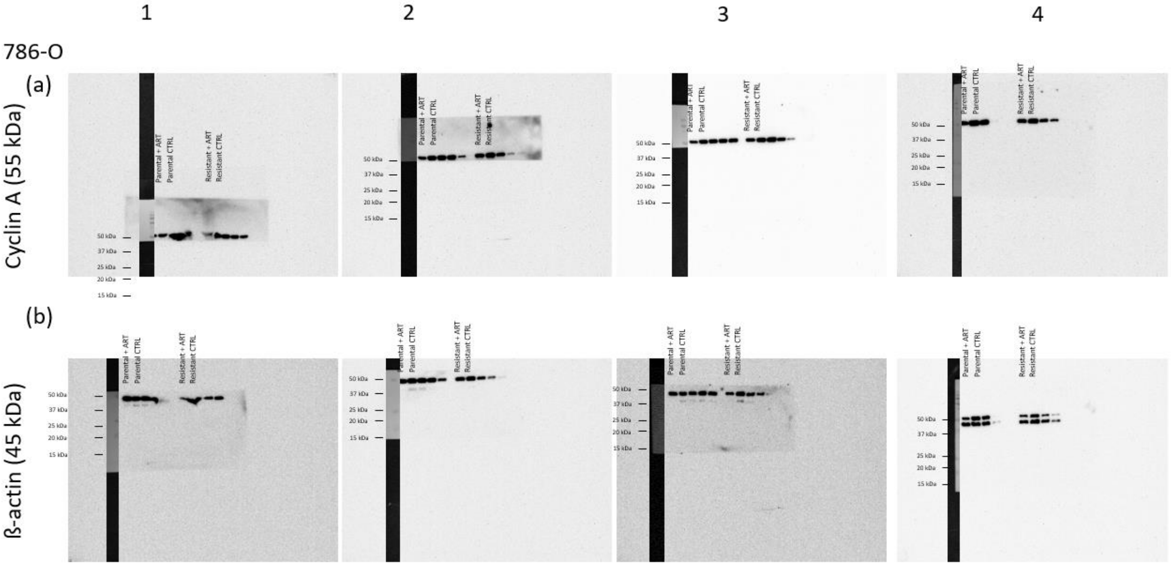
1
2
3
4
786-O
(a)
Cyclin A (55 kDa)
(b)
ß-actin (45 kDa)
Parental + ART
Parental CTRL
Resistant + ART
Resistant CTRL

(f)

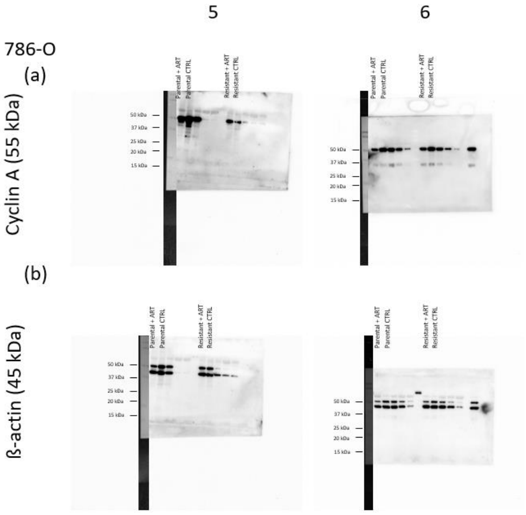
5
6
786-O
(a)
Cyclin A (55 kDa)
(b)
ß-actin (45 kDa)
Parental + ART
Parental CTRL
Resistant + ART
Resistant CTRL

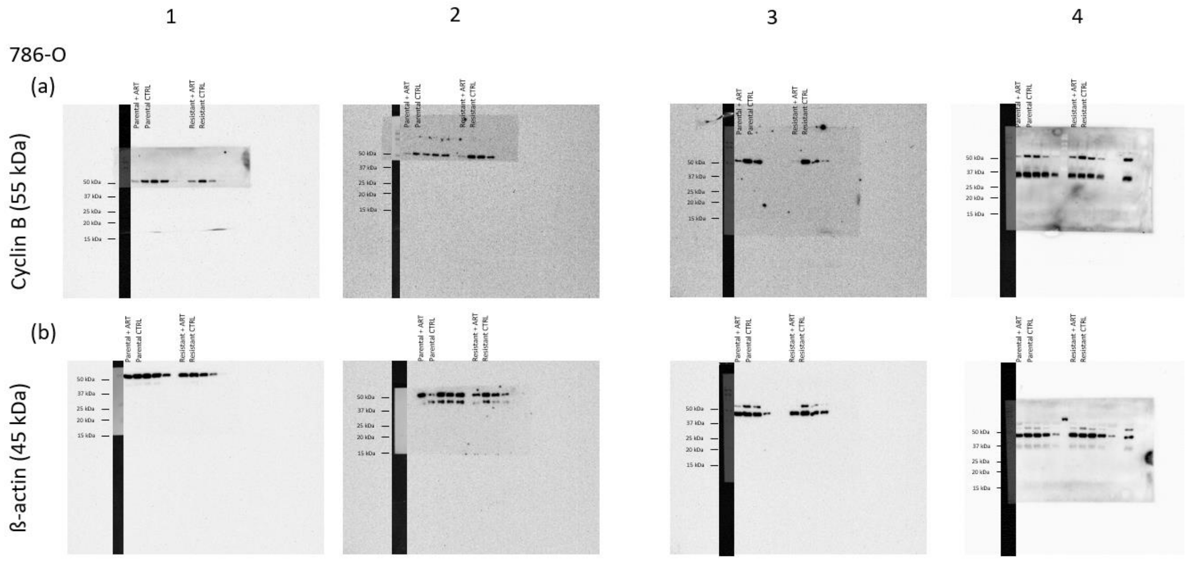

(g)

Figure S2. Detailed information about Figure 7 - Protein expression profile of cell cycle regulating proteins in parental and resistant 786-O cells.

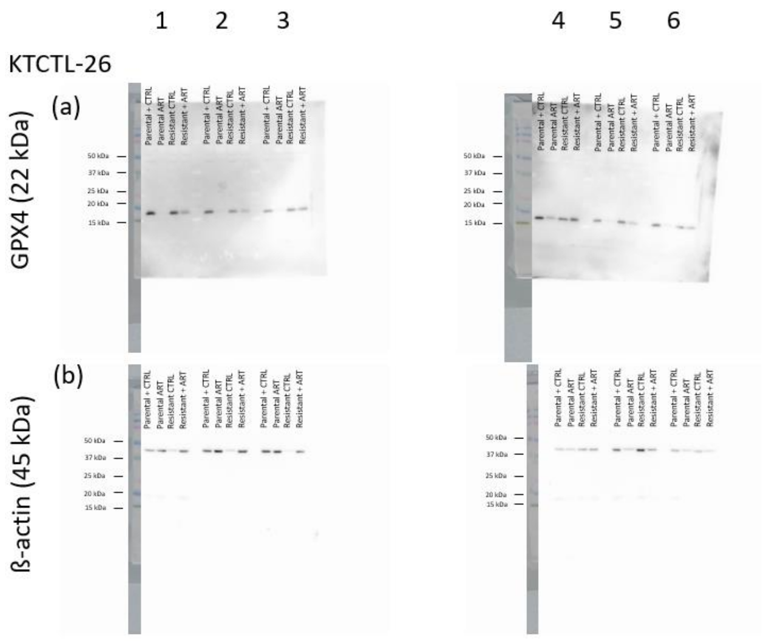

Figure S3. Detailed information about Figure 9f - GPX4 expression in parental and sunitinib-resistant KTCTL-26 cells. Protein expression of GPX4 (**a**), corresponding protein expression of ß-actin (**b**).

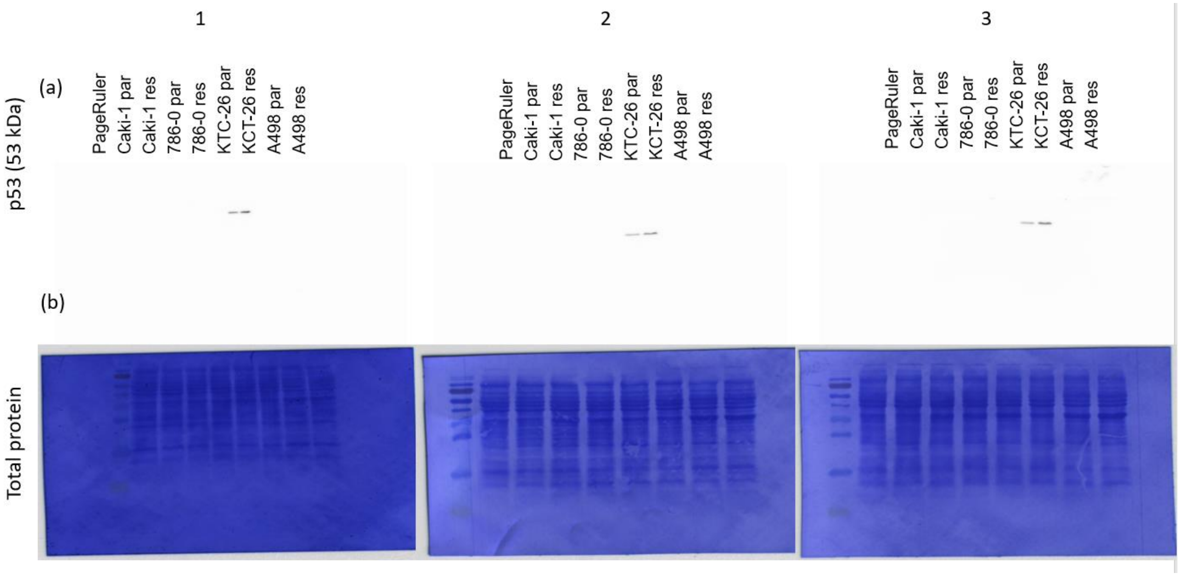

Figure S4. Detailed information about Figure 9h - Protein expression of p53 in parental and resistant Caki-1, 786-O, KTCTL-26, and A-498 cells. Protein expression of p53 (**a**), corresponding Coomassie blue staining of total protein (**b**).

© 2020 by the authors. Licensee MDPI, Basel, Switzerland. This article is an open access article distributed under the terms and conditions of the Creative Commons Attribution (CC BY) license (http://creativecommons.org/licenses/by/4.0/).
